# Generation and characterisation of scalable and stable human pluripotent stem cell-derived microvascular-like endothelial cells for cardiac applications

**DOI:** 10.1007/s10456-024-09929-5

**Published:** 2024-05-22

**Authors:** Qasim A. Majid, Bishwa R. Ghimire, Bela Merkely, Anna M. Randi, Sian E. Harding, Virpi Talman, Gábor Földes

**Affiliations:** 1 National Heart and Lung Institute, Faculty of Medicine, Imperial College London, London, W12 0NN UK; 2 Drug Research Programme, Division of Pharmacology and Pharmacotherapy, Faculty of Pharmacy, University of Helsinki, Helsinki, Finland; 3 Institute for Molecular Medicine Finland (FIMM), Helsinki Institute of Life Science (HiLIFE), University of Helsinki, Helsinki, Finland; 4 MediCity Research Laboratory, University of Turku, Turku, Finland; 5 Heart and Vascular Center, Semmelweis University, 68 Varosmajor Street, Budapest, H1122 Hungary

**Keywords:** Cardiac microvascular disease, Human pluripotent stem cell (hPSC)-derived endothelial cells, Microvascular endothelial cells, Endothelial to mesenchymal transition, 3D cell culture, Vascular organoids

## Abstract

Coronary microvascular disease (CMD) and its progression towards major adverse coronary events pose a significant health challenge. Accurate in vitro investigation of CMD requires a robust cell model that faithfully represents the cells within the cardiac microvasculature. Human pluripotent stem cell-derived endothelial cells (hPSC-ECs) offer great potential; however, they are traditionally derived via differentiation protocols that are not readily scalable and are not specified towards the microvasculature. Here, we report the development and comprehensive characterisation of a scalable 3D protocol enabling the generation of phenotypically stable cardiac hPSC-microvascular-like ECs (hPSC-CMVECs) and cardiac pericyte-like cells. These were derived by growing vascular organoids within 3D stirred tank bioreactors and subjecting the emerging 3D hPSC-ECs to high-concentration VEGF-A treatment (3DV). Not only did this promote phenotypic stability of the 3DV hPSC-ECs; single cell-RNA sequencing (scRNA-seq) revealed the pronounced expression of cardiac endothelial- and microvascular-associated genes. Further, the generated mural cells attained from the vascular organoid exhibited markers characteristic of cardiac pericytes. Thus, we present a suitable cell model for investigating the cardiac microvasculature as well as the endothelial-dependent and -independent mechanisms of CMD. Moreover, owing to their phenotypic stability, cardiac specificity, and high angiogenic potential, the cells described within would also be well suited for cardiac tissue engineering applications.

**Supplementary Information:**

The online version contains supplementary material available at 10.1007/s10456-024-09929-5.

## Introduction

Cardiac microvasculature has long been understudied, and its involvement in cardiac disease overlooked. This is in part due to the inability of conventional angiography to visualise the microvasculature [1]. However, the advent and adoption of advanced, non-invasive cardiac imaging modalities has helped illustrate the significance of this network and how it is impinged upon by cardiac risk factors and disease states, including COVID-19 [2]. Such factors elicit endothelial-dependent and -independent mechanisms that perturb blood flow through the microvessels (arterioles, capillaries, and venules), thus disturbing oxygen supply to the myocardium. This microvascular dysfunction, termed coronary microvascular disease (CMD), may initially manifest as microvascular angina [3] but can proceed to major adverse cardiac events (MACE), including myocardial infarction with non-obstructive coronary artery disease [4] or heart failure with preserved ejection fraction (HFpEF) [1]. Intriguingly, women, as well as non-elderly individuals, are at a greater risk of MACE arising from CMD [4, 5].

Robust in vitro models of the cardiac microvasculature would aid the understanding of these sex differences and allow for the development of treatments for CMD, thereby decreasing incidences of subsequent MACE. However, attaining a suitable population of endothelial cells (ECs) has proven challenging, given the complexity and heterogeneity of ECs. The isolation of native adult cardiac microvascular ECs often requires invasive tissue biopsies, many of which are obtained during vascular surgeries [6] or from failing hearts following transplantation and are, therefore, representative of a disease phenotype. Human microvascular ECs (HMVECs) may be derived with relative ease from the dermis; however, these cells fail to encompass the considerable heterogeneity unique to the organ in which the ECs reside and the tissue-specific angiocrine factors they generate [7].

Human pluripotent stem cell-derived ECs (hPSC-ECs) offer an attractive alternative to native adult ECs. These cells have been reported to express pan-endothelial markers, grow as homogenous cell monolayers that display the traditional cobblestone morphology, and form vessel-like networks both in vitro and in vivo when supported by a matrix [8, 9]. However, during long-term in vitro culture, these cells undergo senescence and demonstrate a decreased propensity for both capillary sprouting and branch point formation [10]. Further, earlier protocols were prone to transdifferentiation into non-endothelial lineages via endothelial-to-mesenchymal transition (EndMT), ultimately limiting their suitability as a stable cell line for disease modelling and cardiac tissue engineering (CTE) applications. Protocols striving for longer-term stability of hPSC-ECs have investigated the preferential selection of an endothelial subpopulation [11] or the inhibition of the TGFβ2-ALK5 signalling axis that has been implicated in EndMT [12]. Yet, they have failed to deliver long-term stability of these endothelial-like cells hPSC-ECs.

These protocols are often comprised of adherent cultures that utilise the tumour-derived Matrigel, which exhibits inherent batch-to-batch variability and, consequently, imparts varied signals between different hPSC differentiations [13]. Moreover, these 2D protocols overlook the mechanical attributes inherent to the 3D environment, as well as the intricate cellular organisation within this spatial context. Therefore, a Matrigel-independent 3D organoid culture protocol, adept at recapitulating the in vivo milieu of the cardiac microvasculature, holds the potential to yield superior vascular cells compared to those obtained through conventional 2D protocols.

More recently, protocols have reported the generation of tissue-specific hPSC-ECs, including cardiac valve hPSC-ECs [14] and brain microvascular-like ECs derived from pluripotent sources [15]. However, RNA-sequencing studies of the latter revealed a lack of traditional endothelial markers and, instead, a transcriptional profile akin to epithelial cells [16]. As such, the production of specified hPSC-ECs would benefit from in-depth transcriptomic and functional analysis to confirm their cellular identity.

Here, we utilised mRNA microarrays and single cell-RNA sequencing (scRNA-seq) to investigate and characterise hPSC-ECs emerging from 2D-monolayers and 3D-vascular organoids, respectively. We found that when treated with high concentration VEGF-A, hPSC-ECs emerging from the vascular organoids were phenotypically stable, expressing markers of human cardiac microvascular ECs. scRNA-seq of the non-EC population contained within the vascular organoid revealed a gene expression profile characteristic of cardiac pericytes, thereby also providing tools for future assessment of the endothelial-independent drivers of CMD. Taken together, we report a stable and well-characterised hPSC-organoid model of the human cardiac microvasculature that can be attained at scale, thus holding great potential for both disease modelling and CTE applications.

## Results

### 2D hPSC-ECs lose their endothelial identity during longer-term culture

**Fig. 1:**
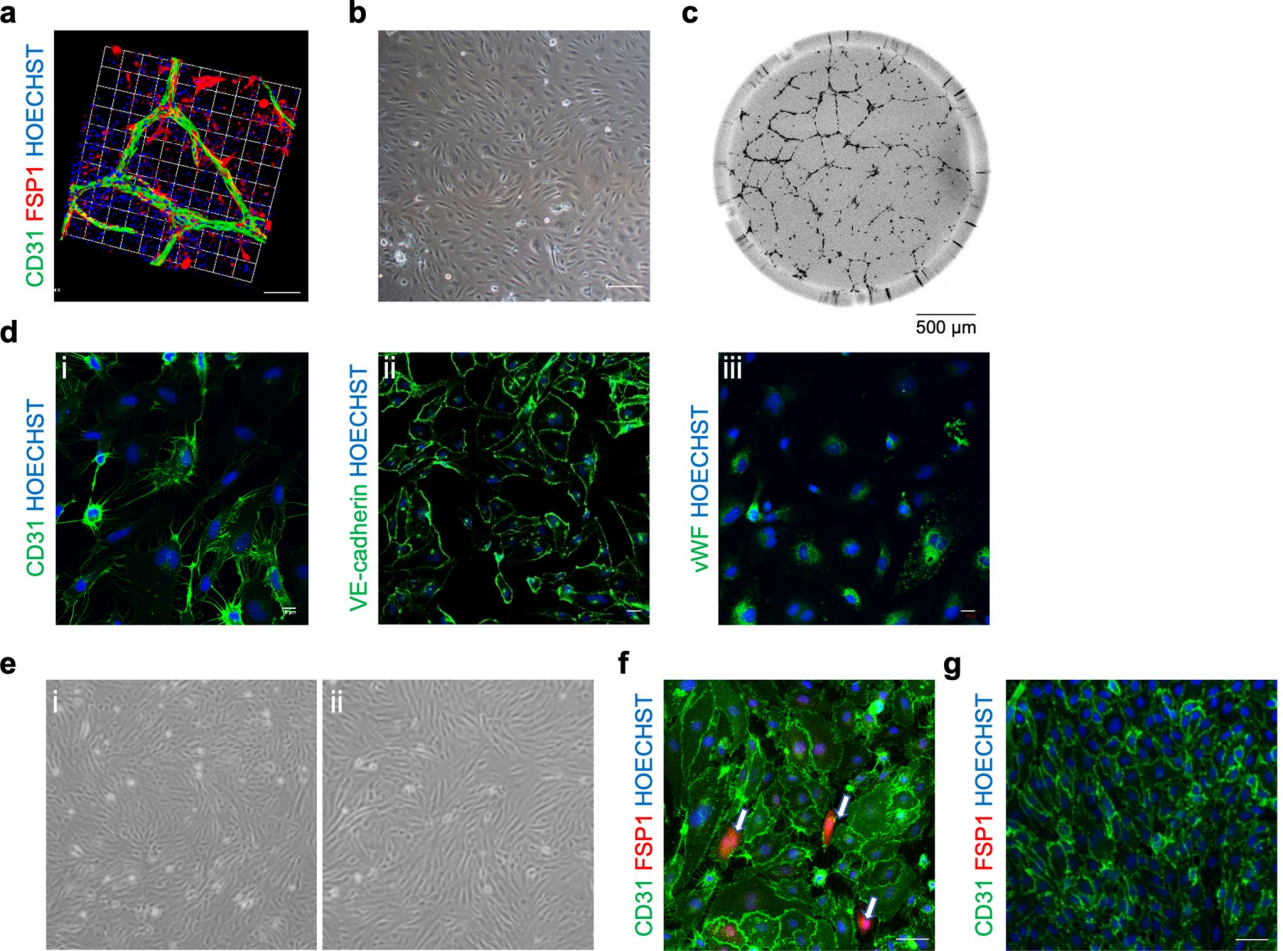
hPSC-ECs attained via a 2D endothelial differentiation protocol results in a heterogeneous population: (**a**) 3D rendering of day 12 2D hPSC-ECs (CD31, green) generating a primitive vascular network supported by non-endothelial cell populations (FSP1, red). Scale bar represents 100 μm. Phase-contrast microscopy images of day 19 2D hPSC-ECs demonstrating (**b**) the classical endothelial cobblestone morphology in adherent monolayer culture and (**c**) in vitro tube formation capacity on growth factor-reduced Matrigel. Scale bar represents 500 μm. (**d**) Representative immunofluorescent images of day 19 2D hPSC-ECs stained for (**i**) CD31, (**ii**) VE-Cadherin, and (**iii**) von Willebrand factor, vWF. Scale bar represents 10 μm. (**e**) Phase-contrast microscopy images of (i) passage 3 and (ii) passage 5 2D hPSC-ECs displaying the emergence of mesenchymal-like cells. Representative immunofluorescent images of P3 (**f**) 2D hPSC-ECs and (**G**) HUVECs cultured for 7 days. Cells were stained for CD31 (green), FSP1 (red), and Hoechst (blue). The white arrows highlight the emergence of the 2D hPSC-EC CD31^pos^/FSP1^pos^ cells undergoing EndMT. Scale bars represent 50 μm

hPSC-ECs were initially derived via a published 2D-adherent growth factor-based protocol [11]; this resulted in a primitive vascular network supported by non-ECs (Fig. 1A). CD31^pos^/NRP1^pos^ hPSC-ECs were purified from the differentiation culture via fluorescence-activated cell sorting (FACS) and cultured in 2D, resulting in the adoption of the traditional endothelial cobblestone morphology (Fig. 1B), formation of tube-like structures in vitro (Fig. 1C), and expression of pan-endothelial marker proteins (Fig. 1D). The expression of endothelial- and mesenchymal-associated genes alongside endothelial transcription factors that are active during embryonic development was assessed by RT-qPCR at three time points during differentiation: day 5 (early differentiation), day 12 (pre-FACS), and day 19 (post-FACS expansion). Although *CD31* expression was upregulated following FACS (Fig. S1A), the expression of mesenchymal markers remained consistently high (Fig. S1B), suggesting that the emerging 2D hPSC-ECs, and not just the supporting non-ECs, expressed mesenchymal-associated genes. Expression of *ETV2*, the master regulator of EC development, displayed a trend towards downregulation, whereas *ERG* and *FLI1*, both involved in endothelial lineage maintenance [17], and *SoxF* transcription factors were upregulated as the 2D differentiation ensued (Fig. S1C-D).

Despite the previously reported long-term in vitro stability of this CD31^pos^/NRP1^pos^ double-positive subpopulation [11], elongated, mesenchymal-like cells began to emerge at passage 3 (Fig. 1Ei), disturbing the cobblestone morphology observed in freshly sorted cells (Fig. 1B). These mesenchymal-like cells proceeded to become the predominant cell type by passage 5 (Fig. 1Eii). This was driven by CD31^pos^/FSP1^pos^ double-positive 2D hPSC-ECs (Fig. 1F) that were absent from both the starting culture and from passage-matched native ECs (Fig. 1G). It is plausible that these cells behave as an intermediary population of 2D hPSC-ECs undergoing EndMT. This was substantiated by the continual downregulation of both *CD31* (Fig. S1E) and the pro-maintenance TGFβ receptor, *ALK1* (Fig. S1F) in passaged 2D hPSC-ECs. Expression of *TGFB2* remained largely unchanged (Fig. S1G); however, downregulation of *ALKI* may suggest a shift towards TGFβ2-mediated EndMT.

### SOX7 is upregulated in 2D hPSC-ECs relative to native ECs

**Fig. 2:**
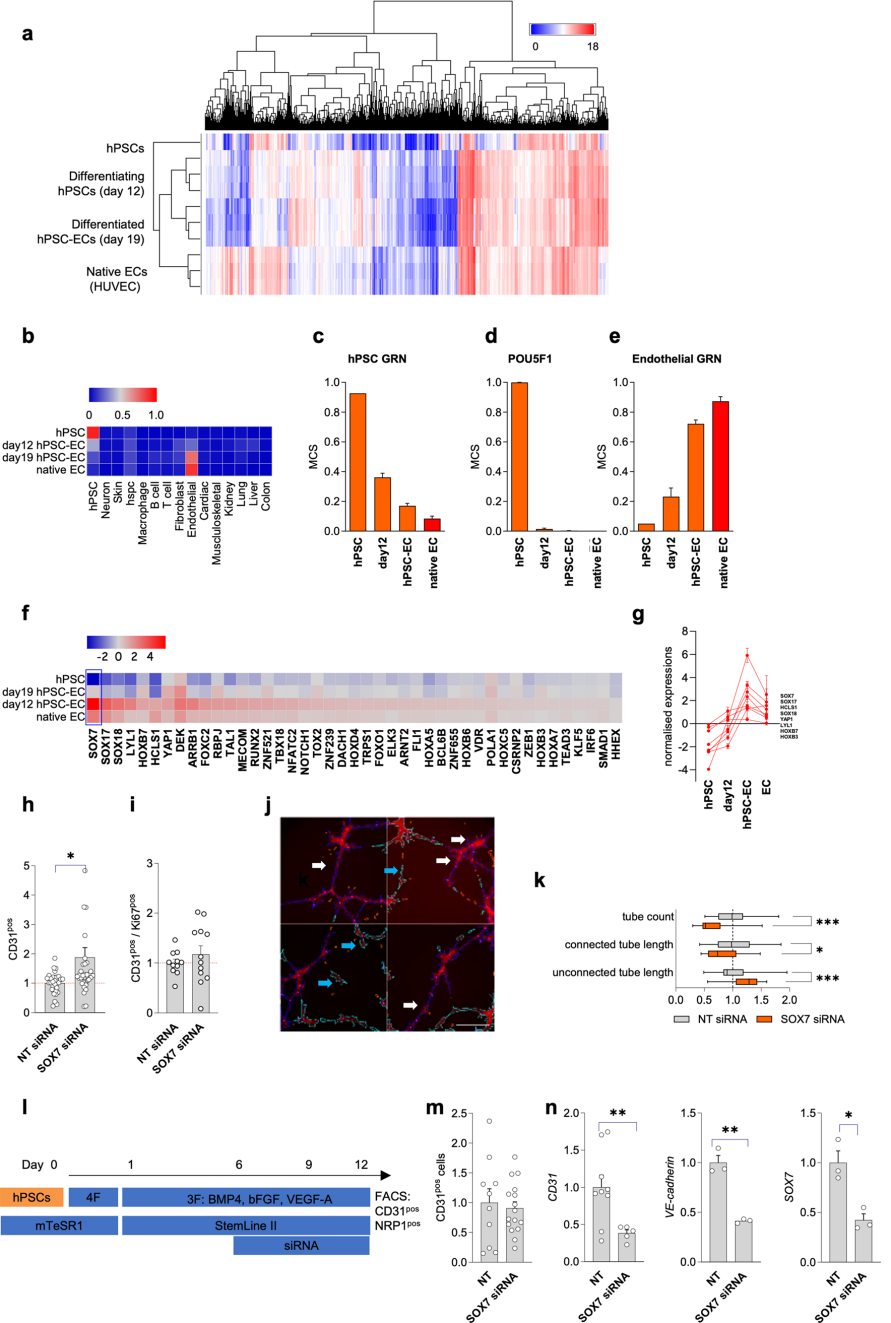
2D hPSC-ECs do not fully engage the endothelial gene regulatory network and have greater *SOX7* expression: (**a**) mRNA microarray-based expression of the four different groups of cells (undifferentiated hPSCs, day 12 2D hPSCs, day 19 2D hPSCs, and HUVECs) (**b**) Heat map displaying high network influence scores as determined by CellNet analysis of microarray data. Each row represents a single cell or tissue type, and each column represents the four different groups of cells. (**c**) Histogram displaying the classification scores of the four different groups of cells for the hPSC gene regulatory network (GRN). (**d**) mRNA levels for the pluripotency marker *POU5F1* as assessed by RT-qPCR for the four cell groups. (**e**) Histogram displaying the classification scores of the four different groups of cells for the endothelial GRN. (**f**) Prioritisation of candidate factors, as identified by CellNet network analysis, that were differentially expressed between the four groups of cells. (**g**) RT-qPCR substantiation to determine expression levels of transcription factor encoding genes (*SOX7, SOX17, HCLS1, SOX18, YAP1, LYL1, HOXB7*, and *HOXB3*) that had been identified as being upregulated in day 19 2D hPSC-ECs relative to the HUVECs. High content image analysis quantification of the fold change of (**h**) 2D hPSC-ECs (CD31^pos^), and (**i**) proliferating 2D hPSC-ECs (CD31^pos^/Ki67^pos^) following culture with either scrambled non-targeting (NT) or *SOX7*-targeted siRNA. *N* = 4–6 independent experiments, unpaired t-test: * *P* < 0.05. (**j**) Representative immunofluorescent image of a 2D hPSC-EC tube formation assay following treatment with *SOX7*-targeted siRNA. Cell viability as assessed by TMRM (red), connected tubes (white arrows), unconnected tubes (blue arrows), scale bar represents 50 μm. (**k**) Quantification of tube area and the length of connected and unconnected tubes formed by 2D hPSC-ECs following *SOX7*-targeted siRNA, *N* = 4 independent experiments. (**l**) Schematic representation of the siRNA treatment regime from days 6–12 during the 2D differentiation protocol. (**m**) High content image analysis quantification of 2D hPSC-ECs (CD31^pos^) expressed as fold change following either NT or *SOX7*-targeted siRNA. (**n**) RT-qPCR of *CD31, VE-cadherin*, and *SOX7* following either NT or *SOX7*-targetted siRNA. Gene expression was determined relative to the NT siRNA group, and data is represented as mean ± SEM, *N* = 4 independent experiments. Target genes were normalised to the housekeeping gene, *GAPDH*. One-way ANOVA with Tukey’s post-hoc test: *P < 0.05; **P < 0.01; ***P < 0.001

mRNA microarray analysis was conducted on 2D hPSC-ECs to ascertain their global gene expression and determine how this differed from native ECs (Fig. 2A). Owing to their longstanding use in endothelial research, human umbilical vein ECs (HUVECs) were utilised as the native population. CellNet analysis of the microarray data highlighted the transition from gene regulatory networks (GRNs) associated with hPSCs to those associated with ECs (Fig. 2B) following endothelial differentiation. GRNs for other mesodermal-derived cells (cardiac and musculoskeletal) were not activated. The mean classification score (MCS, the probability of the cells expressing genes at a level that is indistinguishable from their reference cell type) for hPSCs decreased during endothelial differentiation (Fig. 2C) as did gene expression of the pluripotency marker gene *POU5F1* (Fig. 2D). Although activated, 2D hPSC-ECs had a lower MCS than HUVECs (0.72 versus 0.87, respectively. Figure 2E) suggesting only partial activation of the endothelial GRN. CellNet network analysis also identified candidate transcription factors differentially expressed between the four groups of cells (Fig. 2F). Subsequent RT-qPCR analysis substantiated the upregulation of seven genes, *SOX7, SOX17, SOX18, YAP1, LYL1, HOXB7*, and *HOXB3* (herein, CellNet-identified genes), in D19 2D hPSC-ECs relative to HUVECs (Fig. 2G). With a view to fully recapitulating the native EC GRN, 2D hPSC-ECs were subjected to small interfering RNA (siRNA)-mediated downregulation of *SOX7*. This resulted in an augmentation of the CD31^pos^ population (Fig. 2H), which was independent of an increase in the proliferation of CD31^pos^ hPSC-ECs (Fig. 2I). These data suggest that SOX7 knock-out-dependent improvement in cellular composition was driven by enhanced cell stability and retention of the initial endothelial identity rather than instigating proliferation of the CD31^pos^ population. Functional analysis, however, revealed that *SOX7* siRNA-treated 2D hPSC-ECs generated less complex in vitro vascular networks comprised of unconnected tubes (Fig. 2J, K). Targeting *SOX7* during the 2D endothelial differentiation protocol (Fig. 2L) did not affect the relative abundance of 2D hPSC-ECs (Fig. 2M) but rather impinged upon the differentiation process, resulting in downregulation of both *CD31* and *VE-cadherin* (Fig. 2N) thereby suggesting less mature ECs that would therefore not be suitable for either disease modelling or CTE applications.

### Matrigel-free, 3D suspension culture generates vascular organoids at scale

**Fig. 3:**
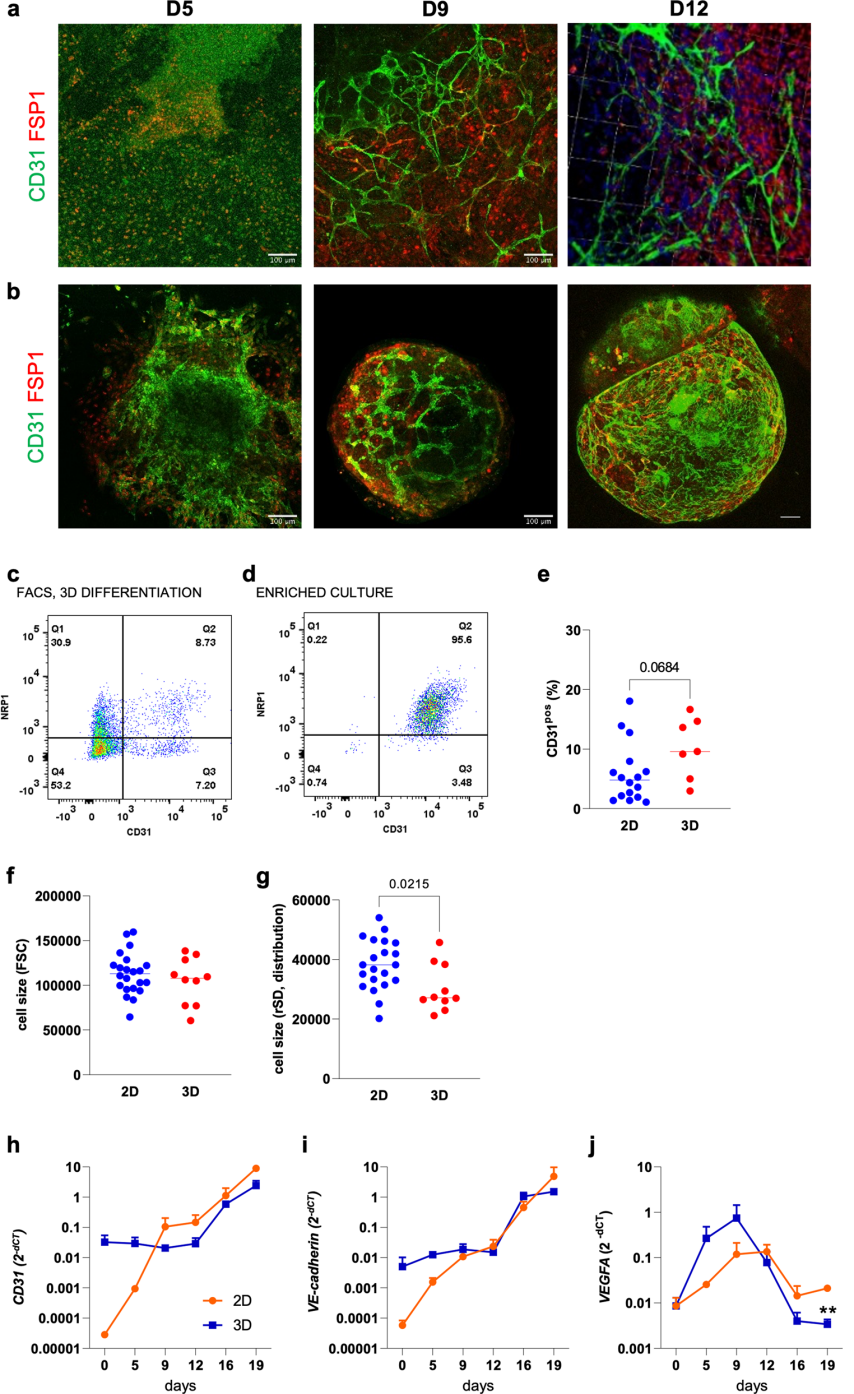
3D endothelial differentiation of hPSCs generates vascular organoids: Representative immunofluorescent images of hPSCs underdoing (**a**) 2D adherent- or (**b**) 3D suspension-endothelial differentiation at days 5, 9, and 12 of the protocol. Scale bars represent 100 μm. (**c**) FACS gating strategy with CD31 and NRP1 expression plotted on the X and Y axis, respectively. FACS gates: Q1: CD31^neg^/NRP1^pos^, Q2: CD31^pos^/NRP1^pos^, Q3: CD31^pos^/NRP1^neg^, Q4: CD31^neg^/NRP1^neg^ cells. (**d**) Validation of endothelial enrichment by flow cytometry. (**e**) The yield of CD31^pos^ hPSC-ECs attained post-FACS is displayed as percentage. *N* = 17 (2D) and *N* = 7 (3D) independent FACS isolations. (**f**) Flow cytometry assessment of cell size from 2D- and 3D-differentiations using the forward scatter (FSC) parameter. (**g**) Homogeneity of cell size generated from either 2D- or 3D-endothelial differentiation as assessed by standard deviation of FSC (rSD). *N* = 21 (2D) and *N* = 10 (3D) independent FACS isolations. Mann-Whitney: *P < 0.05. RT-qPCR of (**h**) *CD31*, (**i**) *VE-cadherin*, and (**j**) *VEGFA* throughout the 2D adherent (orange lines) and 3D suspension culture (blue lines) endothelial differentiation. Target genes were normalised to the housekeeping gene, *GAPDH*. One-way ANOVA with Tukey’s post-hoc analysis: * *P* < 0.05; **P < 0.01; ***P < 0.001

To account for the effects of the 3D microenvironment on the emerging hPSC-ECs, hPSCs were grown as spheroids in an adapted 3D suspension culture protocol devoid of Matrigel. CD31^pos^ cells emerged within these structures by day 5 of the differentiation protocol, forming early vascular networks in contrast to age-matched 2D differentiation (Fig. 3A, B) and continued to develop in complexity throughout the duration of the differentiation protocol. As the CD31^pos^/NRP1^pos^ subpopulation of 2D hPSC-ECs did not confer greater phenotypic stability (Fig. 1F), the total population of CD31^pos^ cells was isolated via FACS and analysed (Fig. 3C, D). Endothelial differentiation of hPSCs in 3D suspension augmented the total number of CD31^pos^ cells with a trend towards a greater yield (*P* = 0.06, Fig. 3E) and generated cells with greater size homogeneity, as assessed via forward scatter analysis (Fig. 3F, G), potentially indicating a more homogenous population of ECs. Despite the absence of Matrigel, 2D- and 3D-hPSC-ECs had comparable gene expression of the pan-endothelial markers (Fig. 3H, I); however, *VEGFA* expression was downregulated in 3D hPSC-ECs (Fig. 3J).

### High concentration VEGF-A treatment facilitates long-term culture stability

**Fig. 4:**
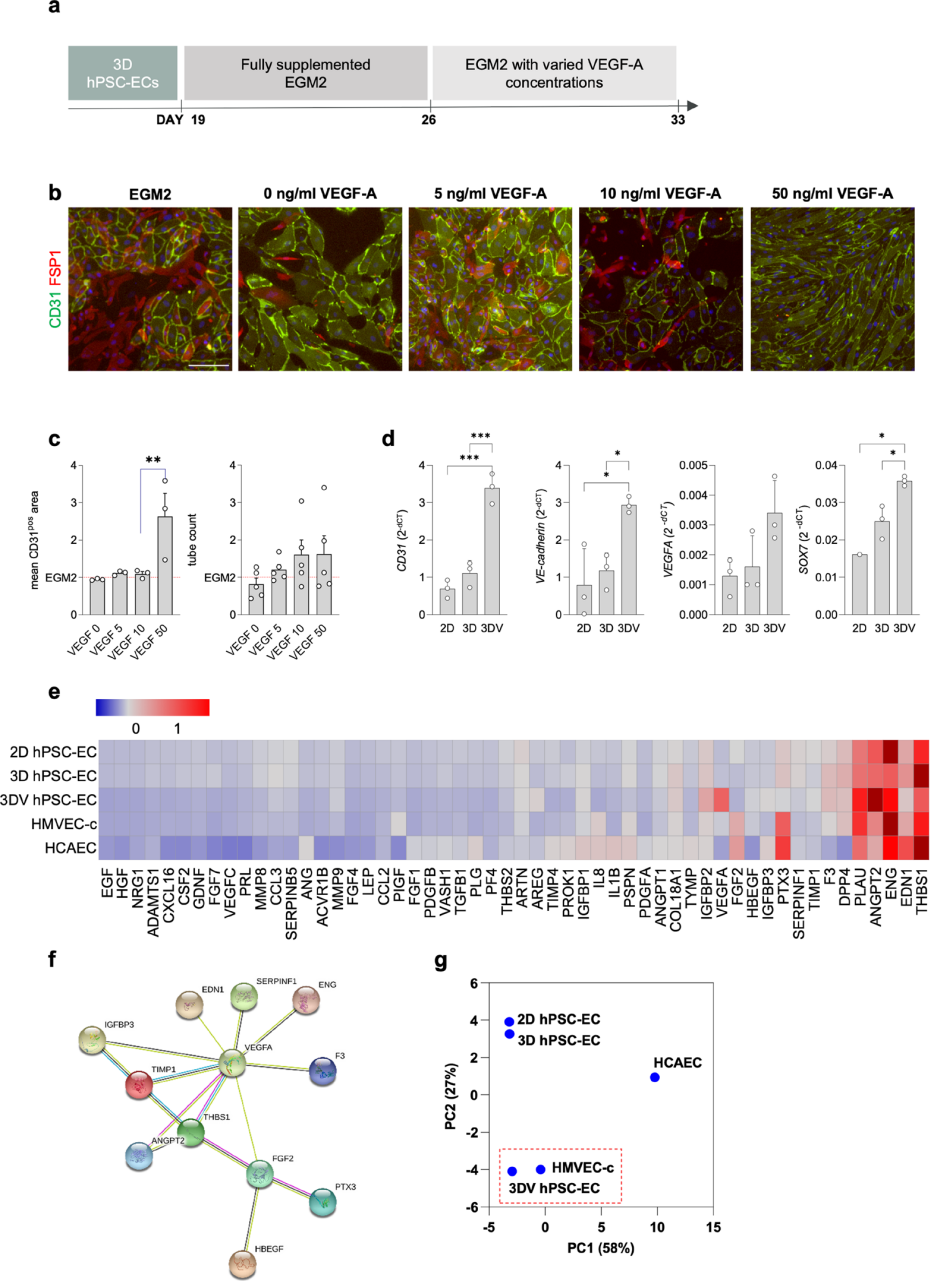
High concentration VEGF-A is required for phenotypic stability of 3D hPSC-ECs: (**a**) Schematic representation of the feeding regime used to establish stable endothelial culture conditions. (**b**) Representative immunofluorescent images of 3D hPSC-ECs maintained under standard culture conditions (EGM2) or varying concentrations of VEGF-A (0, 5, 10, and 50 ng/ml). CD31 (green), FSP1 (red), Hoechst (blue). Scale bars represent 50 μm. High content image analysis for (**c**) mean CD31^pos^ area and number of tubes, expressed as fold change relative to standard culture conditions (fully supplemented EGM2). *N* = 3 independent experiments, one-way ANOVA with Tukey’s post-hoc test: **P < 0.01. (**d**) RT-qPCR analysis of genes associated with endothelial cell identity (*CD31*, *VE-cadherin, VEGFA*) and the vascular endothelial-associated transcription factor *SOX7* as assessed in 2D-, 3D-, and 3DV-hPSC-ECs. *N* = 3 independent experiments, gene expression is expressed as 2^-dCt^, one-way ANOVA with Tukey’s post hoc test: *P < 0.05; ***P < 0.001. (**e**) Heat map displaying the Z score normalised levels of 54 commonly expressed angiogenic proteins in 2D-, 3D-, 3DV-hPSC-ECs, HMVEC-Cs, and HCAECs. (**f**) STRING Protein-Protein Interaction Network analysis of the angiogenic proteins upregulated in 3DV hPSC-ECs. (**g**) Principal component analysis of all 54 angiogenic proteins shows that the 3DV protocol generates hPSC-ECs that have an investigated angiogenic profile highly similar to HMVEC-Cs but not HCAECs

The effects of VEGF-A supplementation on 3D hPSC-EC phenotype and function were evaluated. hPSC-ECs emerging from the 3D differentiation were cultured in either normal EGM2 (herein, 3D hPSC-ECs) or EGM2 supplemented with variable (0, 5, 10, 50 ng/ml) concentrations of VEGF-A (Fig. 4A). Cultures exposed to two weeks of normal EGM2 contained an abundance of mesenchymal cells; however, cultures subjected to increasing VEGF-A concentrations displayed a positive concentration-response to stability, with the highest dose (50 ng/ml) generating homogenous cultures of CD31^pos^ 3D hPSC-ECs (herein, 3DV hPSC-ECs; Fig. 4B). High content image analysis demonstrated a 2.5-fold increase in the mean CD31^pos^ area following high VEGF-A treatment; however, this did not translate to a greater degree of in vitro tubular structures which remained unchanged (Fig. 4C). RT-qPCR analysis revealed 3DV hPSC-ECs had an augmented expression of pan-endothelial markers in addition to *SOX7*, with endogenous *VEGF-A* displaying a trend towards upregulation (Fig. 4D). Moreover, administering 50 ng/ml VEGF-A to 3D hPSC-ECs that had undergone EndMT (Fig. S2A) resulted in the re-emergence of CD31^pos^ cells (Fig. S2B) in these transdifferentiated cultures alongside the restoration of *CD31* and *PNP* gene expression to levels comparable with freshly isolated 3D hPSC-ECs (Fig. S2C-E).

### 3DV hPSC-ECs demonstrate an angiogenic protein profile comparable to that of primary cardiac HMVECs

Comparison of the different hPSC-ECs (2D-, 3D-, 3DV-hPSC-ECs) populations was first conducted at the protein level using a Human Angiogenesis Array immunoassay to determine the expression of 54 angiogenesis-associated proteins. As ECs derived from different vascular networks have unique protein expression profiles, two native EC populations, cardiac HMVECs (HMVEC-Cs) and human coronary artery endothelial cells (HCAECs), representative of the micro- and macro-vasculature, respectively, were used as references of native ECs (Fig. 4E).

Despite receiving high-concentration VEGF-A treatment, the 3DV hPSC-ECs exhibited the highest protein levels of endogenous VEGF-A and angiopoietin-2 (Fig. 4E). This differed from the 3D hPSC-ECs that instead expressed the angiogenesis inhibitor, thrombospondin 1, similarly expressed in HCAECs. Conversely, expression of tissue factor, the key activator of coagulation also implicated in angiogenesis [18], was limited to the groups derived under 3D conditions, indicating that its protein expression was induced independent of exogenous VEGF-A and rather due to signals from the 3D microenvironment. Expression of the endothelial dysfunction-associated protein, Pentraxin 3 [19], was restricted to the native EC populations. Endothelin-1, also implicated in endothelial dysfunction [20], was detected in all EC populations (highest in HCAECs) apart from the 3DV hPSC-ECs.

String protein network analysis of the 3DV hPSC-ECs identified activation of VEGF-related networks with VEGF-A acting as the central mediator (Fig. 4F). Overall, principal component analysis (PCA) conducted for the angiogenic-protein profiles of all EC populations revealed that the 3DV hPSC-ECs exhibited an expression profile similar to that of native HMVEC-Cs (Fig. 4G).

### 3DV hPSC-ECs express markers associated with cardiac microvascular ECs

**Fig. 5:**
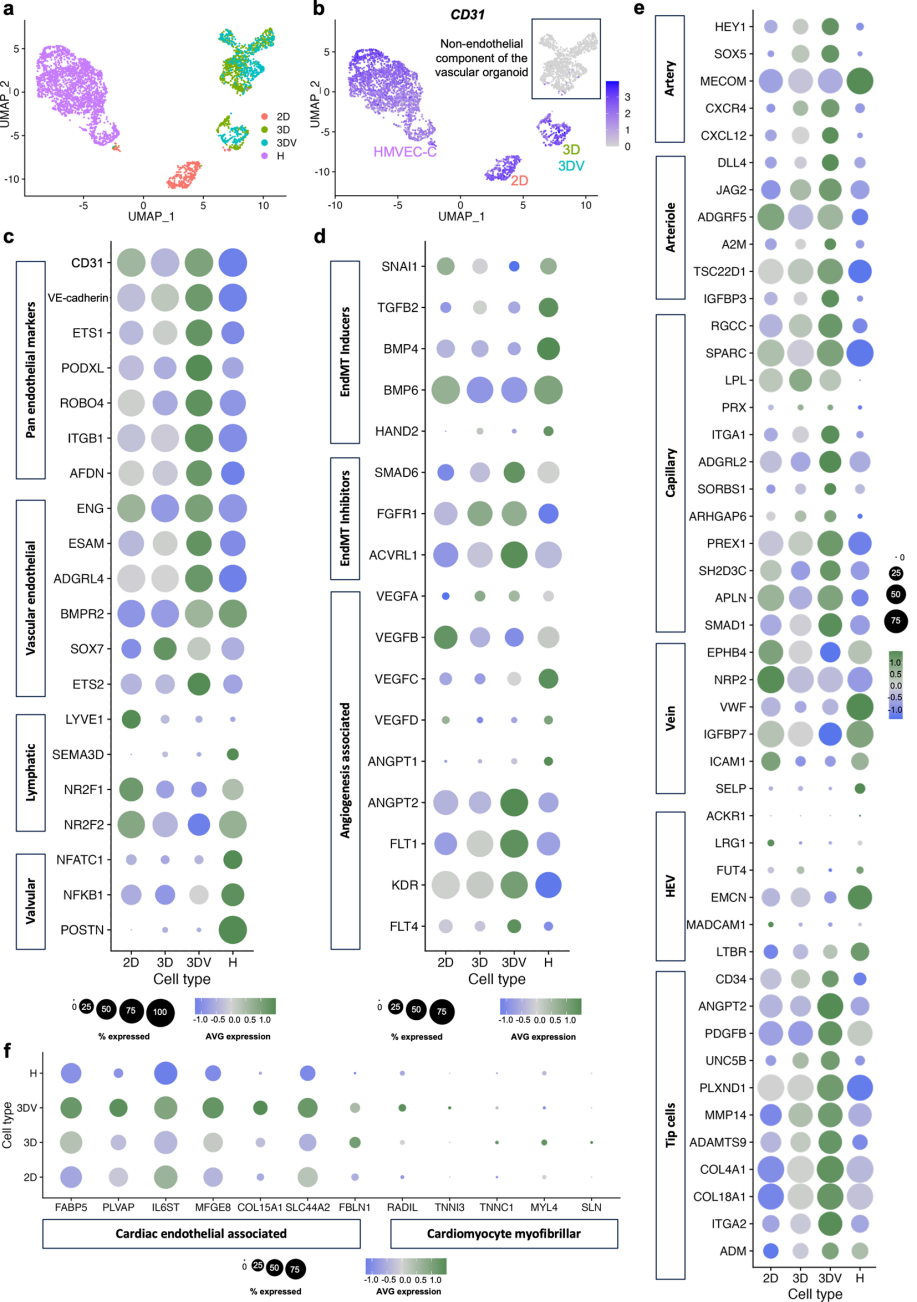
scRNA-seq highlights the cardiac microvascular nature of the 3DV hPSC-ECs: (**a**) Uniform Manifold Approximation and Projection (UMAP) plot showing the integrated analysis of 2D hPSC-ECs, the cells emerging from 3D and 3DV vascular organoids, and HMVEC-Cs via scRNA-seq. (**b**) Feature plot for *CD31* expression. The non-EC (*CD31*^neg^) population arising from the 3D and 3DV vascular organoids is highlighted. Dot plots displaying the expression levels of genes associated with (**c**) pan-, vascular-, lymphatic, and valvular-endothelial cells. (**d**) induction and inhibition of EndMT as well as angiogenesis, (**e**) arteries, arterioles, capillaries, veins, high endothelial venules (HEVs), tip cells and (**f**) cardiac endothelial-associated genes and cardiomyocyte myofibrillar genes, in 2D- 3D-, 3DV-hPSC-ECs, and HMVEC-Cs (referred to as ‘H’). Scales denoting the percentage of cells expressing the gene and the average gene expression are situated below the respective dot plot

Further in-depth characterisation of the different endothelial populations was conducted via scRNA-seq. Despite an apparent similarity in their angiogenic profile, unsupervised clustering following scRNA-seq unveiled that the 3D-derived cells formed two clusters independent from the HMVEC-Cs that were demarcated by their expression of *CD31* (Fig. 5A, B). The *CD31*^pos^ cells were considered hPSC-ECs, whilst the *CD31*^neg^ cells potentially represented the mural cells contained within the vascular organoids. The 2D hPSC-ECs also clustered separately from both the HMVEC-Cs and the 3D-derived cells.

The gene expression of ECs arising from different subtypes, vascular beds, and organs has recently been characterised via scRNA-seq studies [21–25], resulting in the identification of marker genes indicative of diverse endothelial parameters that were evaluated herein. The robust expression of pan-endothelial markers by the 3DV hPSC-ECs substantiated their highly endothelial nature, a stark contrast to the comparatively low expression observed in the HMVEC-Cs (Fig. 5C). Moreover, the 3DV hPSC-ECs preferentially expressed vascular endothelial markers whilst robust expression of lymphatic [25, 26] and valvular [14] endothelial marker genes was identified in the 2D hPSC-ECs and HMVEC-Cs, respectively (Fig. 5C). In addition to *SOX7* expression (Fig. 5C), the 3DV hPSC-ECs expressed several of the CellNet-identified genes at levels greater than the 2D hPSC-ECs (Fig. S3).

In line with their valvular profile (Fig. 5C) and expression of endothelial dysfunction-associated proteins (Fig. 4E), HMVEC-Cs also exhibited prominent expression of genes implicated in EndMT [27–30] (Fig. 5D). Notably, the EndMT-inducing transcription factors *SNAI1* and *BMP6* were also expressed by 2D hPSC-ECs whereas 3DV hPSC-ECs expressed genes associated with the inhibition of EndMT [31–34] (Fig. 5D). Physiological function was also assessed by investigating VEGF-associated genes (Fig. 5D). The VEGF-A protein expression observed in the 3DV hPSC-ECs (Fig. 4E) was corroborated by *VEGFA* gene expression. The 2D hPSC-ECs and the HMVEC-Cs, however, expressed *VEGFB*, associated with pathological angiogenesis [35], with the latter also expressing the lymphangiogenic *VEGFC* and *VEGFD* [36]. Although lacking *VEGFC* expression, 3DV hPSC-ECs expressed its receptor, *FLT4*, in addition to the VEGF-A receptors, *FLT1* and *KDR*.

The vascular bed(s) to which the different EC samples belonged to was then evaluated. As projections of the arteries, arterioles express artery-associated genes in addition to their own specific markers [22, 25, 37] (Fig. 5E). 3DV hPSC-ECs had the highest expression of this gene set with the notable exception of *MECOM*, which is recognised for its role in capillary arterialisation [21], and was instead expressed highly by the HMVEC-Cs. In contrast, *SMAD1* is required for the formation of capillaries [21] and was highly expressed by the 3DV hPSC-ECs alongside capillary-associated genes [24, 25, 38, 39] (Fig. 5E). Conversely, HMVEC-Cs were devoid of these arteriole and capillary markers, thus further questioning their microvascular identity.

Investigation of venous markers revealed preferential expression by the 2D hPSC-ECs and HMVEC-Cs (Fig. 5E). Lymphatic endothelial cells (LECs) are derived from venous ECs during development [40] and therefore share canonical venous markers, including *NR2F2, EPHB4*, and *NRP2* [25, 41, 42]. In contrast to arterioles, fewer venule-specific markers have been established. While the chemokine receptor *ACKR1* has been reported in murine venules [43], its expression was largely absent from all EC populations (Fig. 5E). High endothelial venules (HEV) are specialised post-capillary venules that facilitate the migration of lymphocytes from the bloodstream to lymphatic vessels [44]. Consistent with their lymphatic association (Fig. 5C), 2D hPSC-ECs and HMVEC-Cs also expressed genes associated with the HEVs [45] (Fig. 5E).

In accordance with their capillary-like gene expression profile (Fig. 5E), the 3DV hPSC-ECs were highly enriched for genes associated with tip cells (Fig. 5E) that guide the angiogenic sprout in the direction of the angiogenic stimuli. Tip cells are identified through several markers, including *CD34* [46], and encode genes associated with augmenting angiogenesis [47], metalloproteases for degrading the basement membrane [47, 48], collagen for subsequent basement membrane reconstruction [49], and genes responsible for resolving angiogenic sprouts to prevent aberrant angiogenesis [50, 51]. HMVEC-Cs did not express this gene set, and whilst a subset of these genes exhibited low expression in the 2D- and 3D-hPSC-ECs, the complete profile was robustly detected in the 3DV hPSC-ECs.

In a cardiac context, cardiac microvasculature is essential beyond angiogenesis to maintain cardiac function. Therefore, genes identified in cardiac ECs that mediate such functions were also investigated [52–58]. Indeed, the 3DV hPSC-ECs not only expressed genes associated with coronary angiogenesis [52, 53] but also with cardiomyocyte metabolism [54] and the prevention of EndMT [55, 56]. Further, cardiac ECs also exhibited modest expression of cardiomyocyte myofibrillar genes (CMFs) [59] that were heterogeneously expressed by both 3D-derived hPSC-ECs.

Although the 3DV hPSC-ECs expressed microvascular-associated genes (Fig. 5E) and genes that have been reported to facilitate cardiac function (Fig. 5F), further comparison with *bona fide* human cardiac ECs was required to confirm that high-concentration VEGF-A treatment mediated specification of the 3D hPSC-ECs towards a cardiac microvascular-like profile. The 3DV hPSC-ECs demonstrated robust expression of genes associated with cardiac capillary ECs (Fig. S4A) and cardiac arterioles (Fig. S4B), as identified in healthy adult human cardiac samples [60] and human foetal cardiac ECs [21, 61]. This was in contrast to genes associated with the cardiac macrovessels (Fig. S4C, D) and endocardial cells (Fig. S4E). Transcription factors that are preferentially expressed by different cardiac endothelial subtypes have also been identified from these human samples. To this end, the 3DV hPSC-ECs displayed a profile consistent with cardiac capillary- and microvascular-identified transcription factors (Fig. S4F) yet lacked expression of the cardiac arteriovenous transcription factors (Fig. S4G).

Upon integration with the human samples, the *CD31*^pos^ component of the 3DV-derived cells clustered alongside the *CD31*^pos^ endothelial cells emerging from the human samples whilst displaying divergence from the *LYVE1* expressing cells that were indicative of the human cardiac lymphatic ECs (Fig. S5A-D). The *CD31*^neg^ component of the 3DV-derived cells once again clustered separately (Fig. 5A, Fig. S5A, C) and exhibited pronounced expression of the conventional pericyte marker, *PDGFRB* (Fig. S5E) but not the fibroblast-specific marker, *LUM* (Fig. S6F) that was predominantly derived from the adult human samples. This further strengthened the notion that vascular organoids also generated mural cells that are a critical component of the microvasculature.

Owing to the abundance of non-endothelial cells within this integrated data set (Fig. S5C-E), the ECs were selected for further analysis through their expression of *CD31* and the published data sets were downsampled to attain cell numbers comparable for all samples. The resultant *CD31*^high^ cells from the 3DV hPSC-EC sample (Fig. S6A, B) were characterised by their expression of the capillary marker, *RGCC* [24] and the arteriolar marker *HEY1* [21] whilst lacking expression of *DKK2* [60], that is indicative of cardiac arteries [60] (Fig. S6C-F). This, however, was expressed by the *CD31*^high^ ECs from the human samples alongside *LYVE1* (Fig. S6G) and the endocardial marker, *NRG1* (Fig. S6H). Thus, to conduct a side-by-side comparison of these *CD31*^high^ 3DV hPSC-ECs with their comparative cells from human samples, ECs expressing high *RGCC* (*CD31*^high^/*RGCC*^high^) were ascertained from all samples and were shown to express comparable levels of genes identified in human cardiac microvasculature (Fig. S7).

### 3DV hPSC-ECs upregulate angiogenesis-associated pathways

**Fig. 6:**
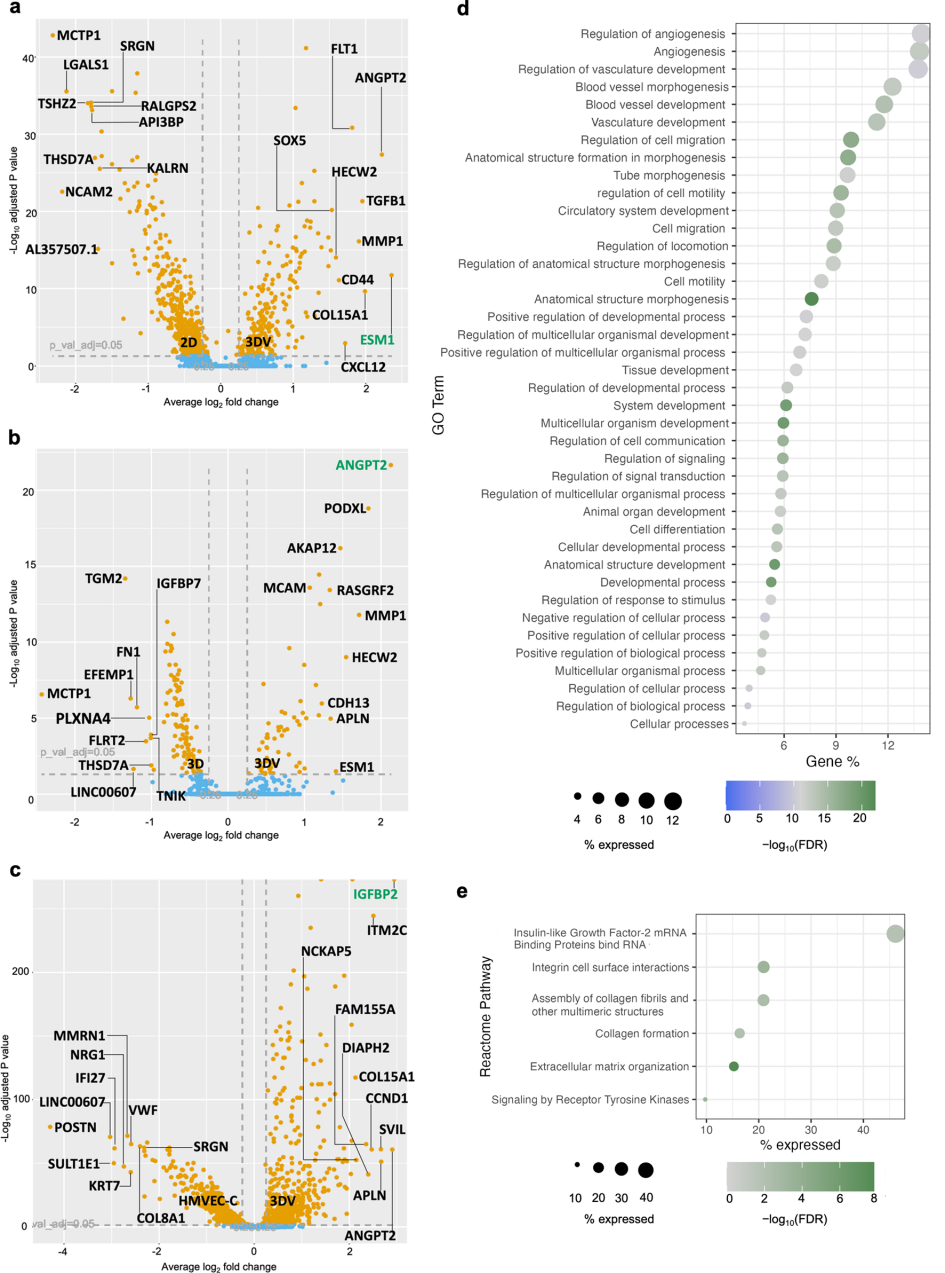
3DV hPSC-ECs exhibit a gene expression profile associated with high angiogenic potential: Volcano plots displaying the top 10 most differentially expressed genes (DEGs), as determined by the highest average log_2_ fold change in 3DV hPSC-ECs relative to (**a**) 2D hPSC-ECs, (**b**) 3D-hPSC-ECs, and (**c**) HMVEC-Cs. The most DEG in the 3DV hPSC-ECs is highlighted in green. (**d**) Gene Ontology (GO) enrichment analysis and (**e**) Reactome Pathway analysis emerging from all of the DEGs identified in 3DV hPSC-ECs relative to HMVEC-Cs. The top 40 GO terms, as determined by their false discovery rate (FDR), are displayed. The scales denote the percentage of genes expressed per GO term or Reactome pathway relative to the reference list for the particular term or pathway and the -log_10_FDR

Unbiased analysis of differentially expressed genes (DEGs) across the four different EC populations (2D-, 3D-, 3DV-hPSC-ECs, and HMVEC-Cs) highlighted the highly angiogenic nature of the 3DV hPSC-ECs. Indeed, the angiogenic-associated genes *ESM1*, *ANGPT2*, and *IGFBP2* [47, 62] were the most upregulated in the 3DV hPSC-ECs relative to 2D hPSC-ECs, 3D hPSC-ECs, and HMVEC-Cs, respectively (Fig. 6A-C). Further, coronary EC markers such as *COL15A1* [53] were significantly elevated in the 3DV hPSC-ECs in comparison to the 3D hPSC-ECs thus further underscoring the role of high VEGF-A treatment in achieving cardiac specificity of these highly angiogenic microvascular hPSC-ECs. In contrast, the 2D hPSC-ECs and HMVEC-Cs upregulated genes associated with LECs [24, 63] and, alongside 3D hPSC-ECs, expressed genes implicated in endothelial dysfunction (*KALRN, POSTN, FN1*, respectively) [64–66]. Gene ontology (GO) enrichment analysis (Fig. 6D) and Reactome Pathway analysis (Fig. 6E) of total DEGs identified in the 3DV hPSC-ECs relative to HMVEC-Cs contextualised this discrepant gene expression profile and further emphasised the highly angiogenic nature of the 3DV hPSC-ECs.

The DEGs identified between 3DV hPSC-ECs and 3D hPSC-ECs were also investigated to further understand the effects of high-concentration VEGF-A treatment on the 3D-derived hPSC-ECs. GO enrichment analysis again demonstrated the highly angiogenic nature of the 3DV hPSC-ECs (Fig. S8A), whilst Reactome pathway analysis revealed activation of the PINCH-ILK-PARVIN pathway (Fig. S8B). *ILK* is expressed in cardiac microvessels where it confers cardioprotection by preventing EndMT [67]. Although HMVEC-Cs also expressed *ILK*, only the 3DV hPSC-ECs expressed *LIMS1* and *PARVA* that complex with *ILK* to form the IPP ternary complex (Fig. S8C) [68]. *ILK* is also implicated in cardiomyogenesis of the human heart and regulates WNT signalling [69]. In turn, the 3DV hPSC-ECs expressed B-catenin (*CTNNB1*) in addition to the cardiac-specific catenin delta 1 (*CTNND*) (Fig. S8E) [61]. Further, *WNT7B* and its receptors (*ADGRA2, FZD4*) were expressed by the 3DV hPSC-ECs. This signalling pathway is implicated in angiogenesis [70] and represses EndMT when in conjunction with *MSX2* [71]. The anti-EndMT capabilities of *WNT7B* are negated by its agonist *DKK1;* however, this was not expressed by the 3DV hPSC-ECs. WNT7B signalling has also been shown to drive the acquisition of blood-brain barrier-like properties in hPSC-ECs [72]; however, the 3DV hPSC-ECs did not mirror this expression profile (Fig. S8F).

### Perivascular cells also emerge from the vascular organoid

**Fig. 7:**
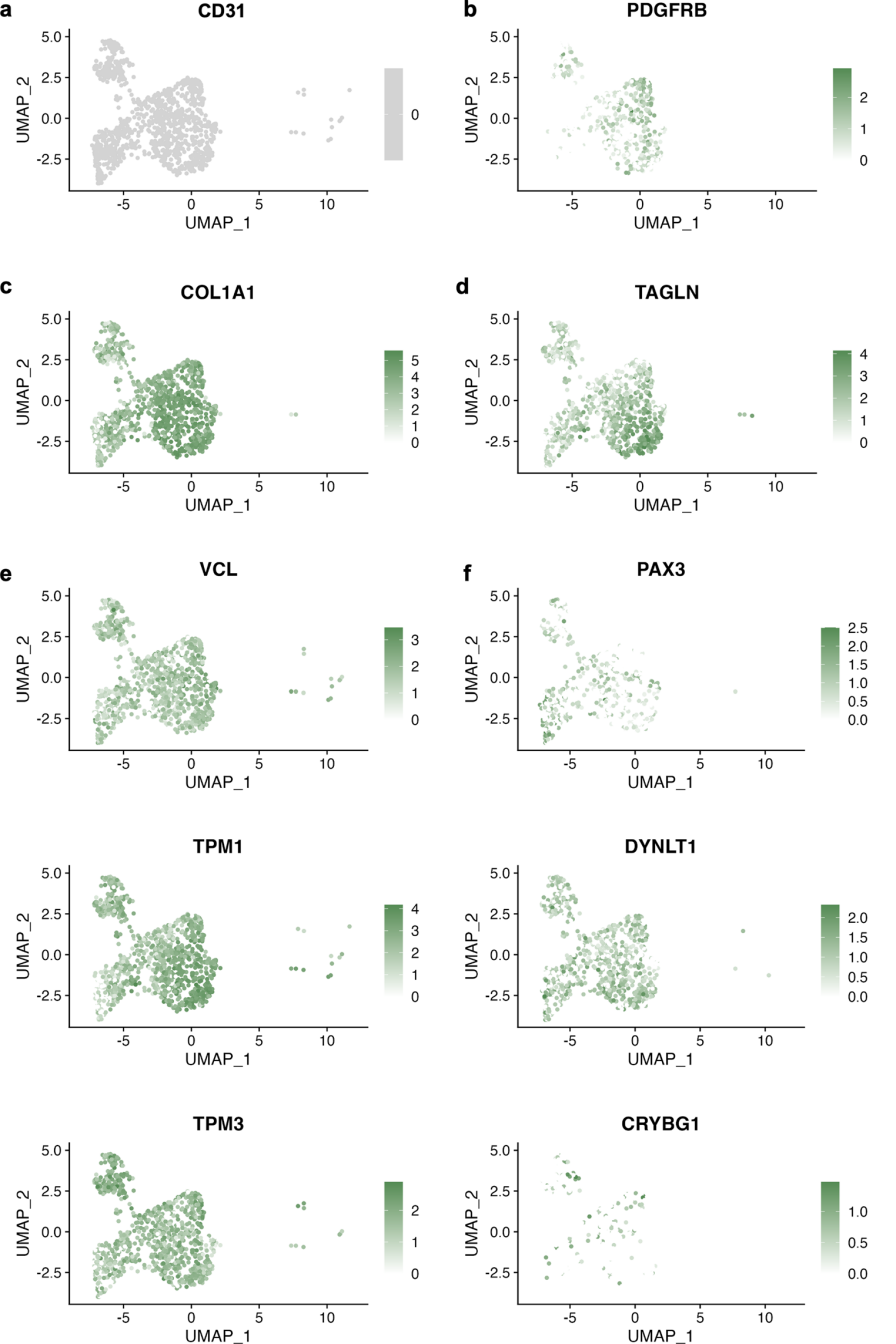
The non-ECs arising from the vascular organoids have a cardiac pericyte-like gene profile: UMAP plots of the *CD31*^neg^ population of cells arising from the vascular organoids showcasing expression of (**a**) *CD31*, (**b**) *PDGFRB*, (**c**) *COL1A1*, (**d**) *TAGLN*, and genes that are (**e**) upregulated in cardiac pericytes (*VCL, TPM1, TPM3*) relative to vascular smooth muscle cells or (**f**) preferentially expressed by cardiac pericytes (*PAX3, DYNLT1, CRYBG1*)

As the *CD31*^neg^ cells from the 3DV-derived cells clustered away from the cardiac fibroblasts and instead with the *PDGFRB*^high^ cells from human cardiac samples (Fig. S5), they were further analysed for pericyte-associated genes. Although this population expressed *PDGFRB* (Fig. 7A, B), it also expressed *COL1A1* and *TAGLN* that have traditionally been considered as fibroblast and vascular smooth muscle cell (vSMC) markers, respectively however, owing to common developmental trajectories of these cells, these are also expressed by pericytes [73, 74]. Further, this population was devoid of recently established fibroblast-specific markers (Fig. S9A) [75] and organ-wide (Fig. S9B) or large vessel-specific (Fig. S9C) vSMC markers [76]. Rather, the *CD31*^neg^ population displayed high expression of genes either upregulated (Fig. 7E) or preferentially expressed by cardiac pericytes relative to vSMCs (Fig. 7F) [77].

The *CD31*^neg^/*PDGFRB*^pos^ subpopulation was also examined and shown to be devoid of markers of non-pericytes (Fig. S10A). Rather, this population demonstrated robust expression of pericyte-associated genes (Fig. S10B) [78] and genes associated with proteins that are secreted by pericytes (Fig. S10C) [79]. Intriguingly, the cells derived from the 3DV culture had lower expression of endogenous *VEGF-A* relative to those arising from the 3D culture, likely due to the abundance of available VEGF-A emerging from the high-concentration treatment. Cardiac pericyte-associated genes were also examined (Fig. S10D), with *RGS4* only expressed by the 3DV population [80, 81]. Further differences between the 3D and 3DV-derived cells were demonstrated by the expression of *CRABP2*, instigated upon cardiac pericyte differentiation towards vSMCs [82], by the former, whilst the 3DV cells exhibited greater expression of ERK1/2 (*MAPK3/1*) that prevents this process (Fig. S10G). Taken together, this population could be isolated from the 3DV cells via FACS for PDGFRβ to attain cells that have a gene expression befitting of cardiac pericytes.

## Discussion

This study describes the development of a scalable 3D protocol enabling the generation of phenotypically stable 3DV hPSC-ECs. Following comprehensive characterisation, we propose designating these cells as cardiac hPSC-microvascular-like ECs (hPSC-CMVECs). In contrast to the previously reported brain microvascular-like ECs derived from pluripotent sources [15, 16], the 3DV hPSC-ECs described in this study displayed high expression of pan-endothelial markers in addition to genes associated with the microvessels, tip cells, and cardiac microvascular ECs. Initial characterisation was performed at the protein level and compared with HMVEC-Cs as a reference cell line. Despite the latter expressing proteins associated with endothelial dysfunction, the principal component analysis suggested an apparent similarity with the 3DV hPSC-ECs. However, scRNA-seq revealed HMVEC-Cs expressed lymphatic and valvular-endothelial cell (VEC) marker genes rather than those associated with the microvessels. This non-microvascular, heterogeneous nature has similarly been reported in dermal HMVECs that exhibited markers of both blood and lymphatic vessels [83]. Alongside this heterogeneity, HMVEC-Cs upregulated genes implicated in the induction of EndMT and endothelial activation. Indeed, periostin (*POSTN*) was the most upregulated gene relative to 3DV hPSC-ECs, and although low-level expression of this complex, multifunctional protein is observed within VECs, it is upregulated during heart failure [84] and can induce the EndMT cascade [85]. These data suggest that these primary cells are not representative of the cardiac microvasculature and are thus not suitable for inclusion within in vitro models of this network.

On the other hand, scRNA-seq of the four endothelial populations assessed alongside a comparison with published data derived from *bona fide* human cardiac samples highlighted the homogeneous, cardiac microvascular nature of the 3DV hPSC-ECs that were devoid of non-vascular markers or markers associated with EndMT and endothelial dysfunction. This refuted the initial similarity postulated between these cells as determined via the Human Angiogenesis Array. This reinforces the need for caution when reviewing and drawing conclusions from highly focused data sets, such as the immunoassay that only considered 54 selected proteins. Nevertheless, *IGFBP2* and *ANGPT2* emerged as the most upregulated genes relative to HMVEC-Cs and 3D hPSC-ECs, respectively, thus aligning with the protein expression profile of the 3DV hPSC-ECs. IGFBP2 augments the transcription of *VEGFA* [86] and thereby may explain the notable protein and gene expression of endogenous VEGF-A in the 3DV hPSC-ECs despite already receiving high-concentration VEGF-A treatment. The effects of this high VEGF-A environment on the resultant endothelial phenotype can be established through a comparison of the 3D-and 3DV-hPSC-ECs.

Both 3D-derived hPSC-EC populations clustered closely together; however, the gene expression profile of 3D hPSC-ECs differed from that of the 3DV hPSC-ECs. Indeed, although a subset of microvascular-associated genes were expressed by the former, their expression levels were markedly lower compared to the 3DV hPSC-ECs. This suggested that the 3D microenvironment of the vascular organoid may be conducive to initiating specification towards the microvasculature but that VEGF-A is required for the completion of this process. As such, the 3D hPSC-ECs may be developmental intermediates that are not yet fully specified and are thus susceptible to EndMT. Evidence of 3DV hPSC-EC cardiac specification is provided by the strong expression of markers identified within *bona fide* cardiac ECs. Intriguingly, whilst the expression of cardiac endothelial-associated genes was restricted to the 3DV hPSC-ECs, the 3D hPSC-ECs had greater expression of the CMF genes. The expression of these canonical CM genes within native cardiac-ECs arises due to their retention of an open chromatin structure around these genes [59]. Helle et al. demonstrated that hPSC-ECs could initiate this gene programme following co-culture with hPSC-CMs [87]. Our cardiac microvascular model was devoid of hPSC-CMs, although it did contain *CD31*^neg^/*PDGFRB*^pos^ that demonstrated a gene expression profile befitting of cardiac pericytes that may similarly induce the expression of CMF genes through chromatin remodelling.

In the context of the microvasculature, pericytes control capillary contractility. Interestingly, these perivascular cells can also serve as progenitors for coronary artery smooth muscle [88] and, as such, share several markers with vSMCs, thus making it challenging to delineate these two cell types. Recent scRNA-seq studies have identified novel gene sets [75–77] that allow for the characterisation of perivascular cells beyond conventional and non-specific markers. By employing these, the non-EC population emerging from the vascular organoids was determined to be in line with that observed for *bona fide* cardiac pericytes. In turn, the inclusion of this cell type within future models of the cardiac microvasculature would allow for both the endothelial-dependent and -independent mechanisms of CMD to be evaluated. This could be particularly valuable, for example, in the investigation into the COVID-mediated dysfunction of cardiac pericytes that leads to microvascular injury and worsened disease prognosis [89].

As with the 3D hPSC-ECs, FSP1 positive cells emerged in FAC-sorted cultures of 2D hPSC-ECs. Endothelial expression of FSP1 is associated with EndMT and driven by *SNAI1* [27], which was highly expressed by the 2D hPSC-ECs and, despite their primary origin, HMVEC-Cs. During development, EndMT is essential for the formation of the cardiac valves [90]; however, its initiation in the adult myocardium is often associated with cardiac fibrosis [91]. Whilst it is plausible that the 2D hPSC-ECs are developmental intermediates undergoing EndMT to complete their developmental trajectory, the expression of genes and proteins associated with endothelial dysfunction suggests these cells are of a diseased phenotype.

CellNet analysis identified the upregulation of several transcription factors in 2D hPSC-ECs relative to the phenotypically stable HUVECs. The most upregulated, *SOX7*, was targeted via siRNA to attain a transcriptomic profile in line with this stable population. However, this resulted in a reduction in *VE-cadherin* expression instead. Intriguingly, the majority of these CellNet-identified transcription factors were upregulated further in the 3D and 3DV hPSC-ECs, suggesting that rather than inducing EndMT, they may be responsible for the cardiac microvascular specification of the ECs. Indeed, in line with our data, *SOX7* has been shown to drive VE-cadherin expression and subsequently inhibit EndMT via this pathway [92]. Moreover, the overexpression of *SOX18* has been shown to enhance the endothelial identity of the iBMECS [93], whilst *YAP1*, through its interaction with *STAT3*, has been shown to mediate VEGF-induced angiogenesis in microvascular ECs [94].

In addition to phenotypically stabilising the 3D-derived hPSC-ECs, 50 ng/ml VEGF-A administration also restored the endothelial phenotype to cultures that had undergone EndMT. Following myocardial infarction (MI), transient activation of a mesenchymal phenotype has been detected within cardiac ECs to potentially aid the re-establishment of the coronary vasculature [95]. A metabolic switch was detected away from traditional cardiac metabolism involving fatty acid binding proteins towards glycolysis in these activated ECs. In our study, 50 ng/ml VEGF-A restored *CD31* and *PNP* (involved in purine metabolism) expression to levels in line with freshly FAC-sorted cells, thus suggesting that the EndMT of these 3D hPSC-ECs may also have been transient. Whilst we believe this process to be driven via VEGF-A, VEGF-B has been implicated in mesenchymal-endothelial transition (MEndoT) that again may be involved in adapting the vasculature to disease [96]. Lineage tracing studies and scRNA-seq of these cultures would be beneficial in establishing the underlying mechanism for this endothelial restoration.

Although the present study provides a comprehensive transcriptomic characterisation of hPSC-derived ECs emerging from scalable 3D culture followed by high-concentration VEGF-A treatment, our study is limited to a single hPSC line. Further investigation is therefore required from 3DV hPSC-ECs emerging from additional hPSC lines as well as diseased hiPSC lines. Moreover, our study largely focuses on a single concentration (50 ng/ml) of VEGF-A; however, other reports have utilised even greater concentrations (100 ng/ml in conjunction with equally high FGF-2) [97]. Thus, it would be intriguing to evaluate whether high-concentration treatment with other growth factors or simply a higher concentration of VEGF-A further influences the specificity and phenotypic maintenance of the hPSC-ECs emerging from 3D culture and, if so, ascertain the pathways responsible for this. At present, we identify the PINCH-ILK-PARVIN pathway in the 3DV hPSC-ECs. Subsequent studies should look to pharmacologically or genetically modulate this pathway to determine its importance in the specification of hPSC-ECs towards a cardiac microvascular-like state. Similarly, although the *CD31*^neg^/*PDGFRB*^pos^ cells demonstrate a gene expression profile befitting of cardiac pericytes, these cells would benefit from further phenotypic and functional characterisation, for example, assessment of their secretome, for which genes have been determined to be expressed by *CD31*^neg^/*PDGFRB*^pos^ cells. Finally, whilst our study presents several facets of evidence investigating EndMT, we must bring attention to the fact that we did not conduct flow cytometric analysis immediately following FACS of the hPSC-ECs. Thus, despite our stringent gating conditions and removal of doublets, the possibility of non-ECs remaining within the sorted cultures cannot be discounted.

In conclusion, we introduce a scalable, hPSC-derived model of the cardiac microvasculature that can be efficiently generated at scale in a Matrigel-independent manner via 3D vascular organoids within stirred tank bioreactors. Upon high-concentration VEGF-A treatment, the emerging endothelial cells exhibit phenotypic stability and distinctly display characteristics of cardiac microvasculature, leading us to suggest the term “hPSC-derived cardiac microvascular-like endothelial cells” (hPSC-CMVECs). Notably, these cells lack markers associated with endothelial dysfunction and instead display a high angiogenic potential, rendering them attractive candidates for inclusion in CTE strategies to repair the post-MI myocardium. Furthermore, when combined with the cardiac pericyte-like cells derived from the vascular organoid, this model presents a valuable tool for exploring the intricacies of the cardiac microvasculature, thus allowing for disease mechanisms to be deciphered and potentially aiding the development of novel therapies aimed at preventing progression towards HFpEF.

## Materials and methods

### Human pluripotent stem cells (hPSCs)

H7 human embryonic pluripotent stem cells were purchased from WiCell Bank (Wi, USA). Growth factor reduced Matrigel (BD Biosciences, US) was diluted 1:50 in KnockOut DMEM (Gibco, UK) and added to tissue culture plastic plates (TCP, Falcon, Corning, USA) that were incubated for 30 min at 37 °C. hPSCs were seeded onto Matrigel-coated plates in mTeSR1 complete medium (Stem Cell Technologies INC, UK) supplemented with 10 µM ROCK Inhibitor Y-27,632 2HCl (Selleckchem, Germany) for 24 h. Culture medium was replaced daily. Cells were subcultured using a 1:3 or 1:6 ratio until 70–80% confluency was reached. For passaging, cells were washed once with PBS w/o Ca^2+^-Mg^2+^ and detached by incubation with Versene solution (Gibco) for 5 min. Cultures were maintained at 37 °C in a 170–300 Galaxy R CO_2_ humidified incubator (RS Biotech), with 5% CO_2_ and 21% O_2_.

### 2D adherent monolayer endothelial differentiation

We optimised a published protocol [11] outlining the 2D adherent monolayer endothelial differentiation of hPSCs. Briefly, hPSCs were detached and collected by centrifugation at 250 g for 5 min. The pellet was resuspended in mTeSR1 media, and hPSCs were replated at a density of 6000/cm^2^ (day − 2). At day 0, the media was replaced with mTeSR1 containing four growth factors: Activin A (R&D systems, UK, #338-AC), bone morphogenic protein 4 (BMP4, R&D systems, #314-PB/CF), basic fibroblast growth factor (bFGF, R&D systems, #4114-TC), and VEGF-A (Peprotech, UK, #100 − 20). All growth factors were used at a concentration of 10 ng/ml throughout the differentiation protocol. After 24 h (day 1), media was replaced with Stemline II hematopoietic stem cell medium (Sigma-Aldrich, UK) containing 3 factors: BMP4, bFGF, and VEGF-A (herein, fully supplemented Stemline II). Samples for RT-qPCR analysis were collected throughout the differentiation protocol. At day 12, CD31^pos^/NRP1^pos^ hPSC-ECs were isolated by FACS, using a FACSAria Cell Sorter (BD Biosciences), and data were analysed using FlowJo software. Briefly, cells were washed in PBS and incubated with 0.05% trypsin-EDTA (TE, Thermo Fisher Scientific, USA) solution at 37 ^o^C for 8 min. Cells were detached mechanically through pipetting, and the TE-cell solution was neutralised in Stemline II media supplemented with 10% foetal bovine serum (FBS, Gibco). Cells were collected by centrifugation at 250 g for 5 min. The cell pellet was resuspended in PBS w/o Ca^2+^-Mg^2+^ supplemented with 1% FBS (FACS blocking buffer). Cells were then incubated with the anti-human CD31 AlexaFluor 488-conjugated (1:20; BD, #557,703) and the anti-NRP1 APC-conjugated antibody (1:11 up to ×10^7^ cells; Miltenyi Biotec, Germany, #130-090-900) diluted in FACS blocking buffer, for 25 min at 4 ^o^C for cell surface double labelling. Single-stained positive controls were generated by incubating with either anti-human CD31 AlexaFluor 488-conjugated or the anti-NRP1 APC-conjugated antibody, and negative controls were derived by incubating cells with FACS blocking buffer only. Cells were then washed in FACS blocking buffer and passed through a 70 μm cell strainer (StarLab, UK) to ensure complete dissociation into single cells prior to FACS. Sorted CD31^pos^/NRP1^pos^ cells were collected in collection media (40% 10 ng/ml factor supplemented Stemline II medium, 40% endothelial growth factor medium-2 (EGM2, Lonza, UK), 19% FBS, and 1% penicillin/streptomycin solution (P/S, Gibco) and seeded at a density of 2500/cm^2^ onto TCP that had been pre-coated with type IV collagen (Sigma-Aldrich, #C7521). Cells were maintained in replating media (50% EGM2, 49% fully supplemented Stemline II, and 1% P/S) for 48 h and thereafter replaced with 75% EGM2, 24% fully supplemented Stemline II, and 1% P/S for a further 48 h. From day 16 onwards, cells were maintained in 100% EGM2 media until they reached 80% confluency, at which point they were detached using 0.05% TE and subcultured (passage 1) at 10,000–12,000 cells/cm^2^ in fully supplemented EGM2 that was replaced every two days.

### Endothelial cell culture conditions

HUVECs were isolated as previously described [98]. Human coronary arterial endothelial cells (HCAECs) and human cardiac microvascular endothelial cells (HMVEC-Cs) were purchased from Lonza. Cells were plated on type IV collagen-coated flasks and cultured in fully supplemented EGM2 with media replaced every two days. Upon reaching confluency, cells were dissociated with 0.05% TE and, split 1:3 and replated onto type IV collagen-coated flasks.

### Real-time quantitative PCR

TriReagent (Invitrogen, US) was used to isolate RNA, and purification was performed using an RNeasy Mini Kit (Qiagen, Germany) following the manufacturer’s instructions. Reverse transcription was carried out by High-Capacity cDNA Reverse Transcription Kit (Invitrogen). Expression of the following genes was determined using TaqMan PCR: (*CD31*: Hs00169777_m1; *VE-cadherin*: Hs00901463_m1; *ACTA2*: Hs00426835_g1; *CNN1*: Hs00959434_m1; *ERG*: Hs01554629_m1; *SOX7*: Hs00846731_s1; *SOX17*: Hs00751752_s1; *SOX18*: Hs00746079_s1; *POU5F1*: Hs00999634_gH; *HOXB3*: Hs05048382_s1; *HOXB7*: Hs04187556_m1; *YAP1*: Hs00902712_g1; *LYL1*: Hs01089802_g1; *VEGFA*: Hs00900055_m1; *PNP*: Hs01002926_m1; *GAPDH*: 4,333,764 F endogenous control, Applied Biosystems, US). TaqMan Universal Mastermix II (Applied Biosystems) was diluted 1:2 and used for all reactions. All assays were diluted 1:20. Samples were run on the Mastercycler RealPlex 2 (Eppendorf, US) Real-Time PCR system. *ETV2, FLI1, ACVRL1, ACTA2*, and *TGFB2* expression levels were determined using an SYBR Green mix (PerfeCTa SYBR green FastMix, Quantabio, US). The SYBR green FastMix was diluted 1:2 and used for all reactions. Forward and reverse primers were used at a concentration of 10 pmol/µl and were diluted 1:25. The sequences of all the primers used for SYBR green reactions are shown in Table 1. Samples were run on the C1000 Thermal Cycler CFX96 Real-Time PCR system (BioRad, US). qPCR reactions were carried out in triplicate for all genes of interest. Relative expression was determined using the ΔΔCt method with GAPDH as the housekeeping control.

### Immunocytochemistry

hPSC-ECs and HUVECs were seeded onto glass coverslips coated with either type IV collagen or 1% gelatine, respectively. These were fixed with 4% PFA at room temperature (RT) for 10 min and permeabilised with 0.2-0.5% Triton X-100 for 5 min. Cells were stained with primary antibodies against CD31 (Alexa Fluor 488-conjugated, BD #557,703, 1:50), VE-cadherin (BD #55,561, 1:250), FSP1/S100A4 (MerckMillipore, US, 07-2274, 1:100), Ki67 (Abcam, UK, ab833, 1:100), and VWF (Dako, Denmark #A0082, 1:1000) for 60 min, at RT. Cells were subsequently washed with PBS and incubated for 45 min with AlexaFluor 488 and 555-conjugated secondary antibodies (Invitrogen #A11008 and #A21424, 1:400) at RT. All antibodies were diluted in PBS containing 3% bovine serum albumin (BSA, Thermo Fisher Scientific). Cells were again washed with PBS to remove unbound secondary antibodies, and coverslips were mounted onto slides using Fluoromount G (Southern Biotech, US). Cell nuclei were visualised using Hoechst 33342 (Thermo Fisher Scientific, #H1399, 1:200).

### Confocal and high-content automated microscopy

Specimens were imaged using confocal microscopy (Zeiss LSM-780 inverted), and data was analysed with ZEN 2012 (Zeiss, Germany) and ImageJ software. For the automated quantification of intensities in each cell compartment, cells seeded in 96-well and 384-well plate formats were imaged using the ArrayScan™ VTi automated microscopy and image analysis system (Thermo Fisher Scientific), using modified Target Activation and Morphology Explorer (for cell area calculation and structural assessment) BioApplication protocol. Cells were identified with Hoechst in channel 1 (excitation 350 nm), CD31 in channel 2 (488 nm), and FSP1 or Ki67 in channel 3 (546 nm). The percentage of cells with high average intensities was quantitated using a 10x objective for cell fluorescence intensity measurement. Live imaging of ECs was achieved following Hoechst and TMRM staining (Thermo Fisher Scientific).

### Matrigel tube-formation assay

Endothelial vascular network formation was assessed in an automated manner in Ibidi 96 well µ-Plates. Growth factor reduced undiluted Matrigel was thawed overnight at 4 °C on ice. Plates were coated with 10 µl Matrigel per well and incubated for one hour at 37 °C. 10,000 2D hPSC-ECs were seeded per well and imaged after 6 and 24 h using a high content microscopy system. Each condition was performed in duplicate. Quantification of tube length and network interconnectivity was performed using the angiogenesis bioassay in ArrayScan VTi.

### Microarray analysis

Total RNA was extracted as described above in triplicate and resuspended in nuclease-free water. The integrity of the extracted RNA was assessed using a Bioanalyser, whereby 5 ng of the sample was used to prepare biotin-labelled cell extract using the Nugen Ovation amplification system. For each representative biological replicate group, 7 µg of the labelled extract was hybridised to Affymetrix 2.0 GeneChips for 20 h. The hybridised arrays were washed, stained, and scanned according to the manufacturer’s instructions. Data from individual GeneChip were MAS5 pre-processed. The signal from every GeneChip was normalised to the median of the signal distribution on that array, followed by individual genes being normalised to the median of the distribution of their signal across the whole experiment. Unreliable gene expression measurements were removed by the application of the Affymetrix Flag filter before any statistical analysis. From these data, genes that did not vary beyond a 2-fold range across the whole experiment were also removed. Prioritised transcriptional regulators were selected based on comprehensive microarray gene expression profiles and subsequent prioritisation with CellNet.

### Silencing of *SOX7*

Small interfering RNA (siRNA) knockdown was performed on differentiating hPSCs and passage 2–3 purified 2D hPSC-EC cultures. Dharmacon SMARTpool: ON-TARGETplus siRNA (25 nM, GE Healthcare, US) was performed as per the manufacturer’s instruction. Scrambled non-targeting (NT) siRNA (Control siRNA ON-TARGETplus Non-targeting Pool 25 nM) was used as a negative control. The MirusBio TransIT-siQUEST transfection reagent was used to transfect cells with the siRNA. Differentiating hESC cultures were re-transfected every 48 h to maintain the effects of siRNA.

### Endothelial differentiation of hPSCs in 3D stirred-tank bioreactors

hPSCs were detached from their maintenance culture as previously described and grown in suspension culture by placing 50 ml mTeSR1 medium containing 10 µM Y-27,632 2HCL into a 100 ml CELLSPIN glass spinner flask (Pfeiffer, Germany). The flask was placed onto the CELLSPIN magnetic stirring system (Pfeiffer, Germany) within an incubator at 37 ^o^C with 5% CO_2_. Following 24 h of continuous stirring, a sample of the cell suspension was aliquoted from the spinner flask, and the presence of cell clusters was checked under a light microscope. If present, media was replaced with fresh mTeSR1 for a further 24 h, at which point endothelial differentiation was induced by adding the growth factors (10 ng/ml) to the media as previously described for the 2D adherent monolayer differentiation. Cell cultures were monitored during the differentiation process for signs of contamination or cell death. On day 12 of the differentiation protocol, cell clusters were dissociated into single cells using the human embryoid body dissociation kit (Miltenyi Biotec) as per the manufacturer’s instructions. Dissociated cells were passed through 70 μm and 40 μm cell strainers (StarLab) to ensure cell clusters were fully dissociated into single cells. Cells were prepared for FACS as previously described. Day 12 CD31^pos^ hPSC-ECs were cultured in type IV collagen-coated T225 flasks until confluency had been reached (day 19).

### Proteome profiling of hPSC-ECs

Angiogenesis proteome profiling was performed on ECs using the Proteome Profiler Human Angiogenesis Array Kit (R&D Systems, ARY007). Sample preparation and experimental setup were performed following the product catalogue guide. The pixel density of the X-ray films was analysed by ImageJ software. Protein-protein interaction networks functional enrichment analysis was performed by String DB (string-db.org). Principal component analysis was performed by Microcal OriginPro 2016.

### Improved maintenance of hPSC-EC long-term culture

Day 19 hPSC-ECs were subjected to a two-week concentration-response using different concentrations of VEGF-A. Cells were cultured in either fully supplemented EGM2 or EGM2 medium prepared by adding all SingleQuots to endothelial basal medium-2 (EBM2) except the VEGF-A SingleQuot. This media was then supplemented with either 0, 5, 10 or 50 ng/ml VEGF-A.

### Single-cell RNA-sequencing

2D, 3D-hPSC-ECs, and HMVEC-Cs were maintained in fully supplemented EGM2, whilst the 3DV hPSC-ECs were cultured in EGM2 containing 50 ng/ml VEGF-A. As the hPSC-ECs and HMVEC-C samples were derived from two separate differentiations or passages, respectively, cells were cryopreserved in liquid nitrogen until all samples had been produced. At this point, the four endothelial populations were removed from liquid nitrogen and thawed at 37 ^o^C for 2 min. The thawed cells were transferred to pre-heated media in a dropwise manner. For each sample, the biological replicates were combined, and cells were centrifuged at 200 g for 5 min. Cell viability was determined via trypan blue staining. The cell pellet was resuspended in PBS containing 1% BSA, and the four samples were loaded into a 10x Genomics microfluidic chip that was placed into a 10x Chromium controller.

scRNA-seq libraries were sequenced using Illumina’s Novaseq 6000 sequencer. Bcl files produced by the sequencer were converted to FASTQ files using Cellranger (version 7.1.0) from 10x Genomics. The same software was used to generate single-cell level count data. Count data from the Cellranger aggregate pipeline was used in downstream data analysis. Downstream analysis was performed in R (version 4.3.0) using the Seurat (version 4.1.3) package [99]. The cells in which fewer than 500 genes were detected and the genes which were not detected in at least 5 cells were discarded. Further, cells with fewer than 1000 and greater than 7000 detected genes were also removed, as were cells with a mitochondrial gene percentage greater than 10%. Normalisation was performed using the SCTransform method. All four samples were combined using Seurat’s integration workflow. Principal component analysis (PCA) was performed using the 2000 most variable genes. The first 30 principal components were used in clustering and UMAP dimensional reduction. Endothelial cells were selected by their expression of the marker gene *CD31*. Differential gene expression analysis was performed between 2D and 3DV, 3D and 3DV, and HMVEC-C and 3DV using the default statistical test, Wilcoxon Rank Sum test. The statistical test was performed only on the genes detected in at least 25% of cells in either of the comparison groups. Differentially expressed genes with an adjusted P-value > 0.05 and absolute(logFC) < 0.25 were removed, whereas genes with an average log_2_ fold change > 0.25 and a P-value < 0.05 were considered differentially expressed. Subsequent GO Enrichment Analysis (conducted via Geneontology.org) and Reactome Pathway Analysis (performed on Reactome.org) incorporated all the identified DEGs between two particular samples. The generated results were organised based on their false discovery rate.

### Data analysis

Statistical analysis was conducted with GraphPad Prism 8 software. All values are stated as mean ± standard error mean (SEM) unless otherwise stated. Statistical significance was calculated using Student’s t-test, one-way ANOVA following Tukey post-hoc test or Mann-Whitney test where appropriate. A P-value of less than 0.05 was considered significant. Corrections for multiple testing were applied when relevant using the Holm-Sidak method. All experiments were performed using at least three biological replicates (N). Screening response statistics used the z-score method to calculate standard deviations from the mean for each data point in a normal population of the data set. The datasets generated during and/or analysed during the current study are available from the corresponding author on reasonable request.

**Table 1:** Sequences of the forward and reverse primers used for the SYBR green reactions to determine the expression of *FLI1, ETV2, ALK1, TGFB2*, and *GAPDH*.

Gene of interest	Forward primer	Reverse primer
FLI1	ACGGGACTATTAAGGAGGCTCTG	GTTGACCCTCACTGGCTGATT
ETV2	ACTAACCACCGAGGTCCCAT	CTGTTGCCAGTCCAACGGAT
ALK1	TGGAGTGTGTGGGAAAAGGC	ATCTCAGTCTCCCGGAACCA
TGFB2	GGTACCTTGATGCCATCCCGCC	GCACTCTGGCTTTTGGGTTCTGCA
GAPDH	CAAGGTCATCCATGACAACTTTG	GGGCCATCCACAGTCTTCTG

### Electronic supplementary material

Below is the link to the electronic supplementary material.

Supplementary Material 1

## Data Availability

The datasets generated during and/or analysed during the current study are available from the corresponding author on reasonable request.
