# Supplementary material for: Generation and characterisation of scalable and stable human pluripotent stem cell-derived microvascular-like endothelial cells for cardiac applications
Source: Angiogenesis. 2024 May 22;27(3):561–82. doi: 10.1007/s10456-024-09929-5 (PMC11303486; doi:10.1007/s10456-024-09929-5)
Supplement: Supplementary file 1 — Supplementary Material 1 [file 10456_2024_9929_MOESM1_ESM.docx]

**Generation and characterisation of scalable and stable human pluripotent stem cell-derived microvascular-like endothelial cells for cardiac applications**

**SUPPLEMENTARY MATERIALS**

**SUPPLEMENTARY METHODS:**

**Comparison of the 3DV-derived cells with published scRNA-seq datasets of human cardiac ECs:** The scRNA-seq data of the two healthy human hearts explored in Koenig AL *et al*. [60] and the 13-week human foetal cardiac EC sample investigated in McCracken IR *et al*. [21] were combined with the 3DV-derived cell sample using Seurat’s (version 4.1.3) integration workflow in R (version 4.3.0) as described earlier in the main methods section. Data were filtered, normalised, and clustered as previously described allowing for visualisation of the complete integrated data set. A secondary, focused data set was derived to assess the ECs within this heterogenous complete integrated data set through the selection of cells with a *CD31* expression greater than 1 (*CD31*^high^). Further, the published samples were randomly downsampled to 1000 random cells to attain cell numbers more comparable to the 3DV-derived cell sample. To assess the microvascular ECs within this secondary dataset, ECs enriched for the capillary marker, *RGCC* (expression greater than 1: *CD31*^high^/*RGCC*^high^) were selected and utilised for side-by-side comparison of cardiac endothelial genes within the 3DV hPSC-ECs and the *bona fide* human cardiac ECs.

**SUPPLEMENTARY FIGURES:**


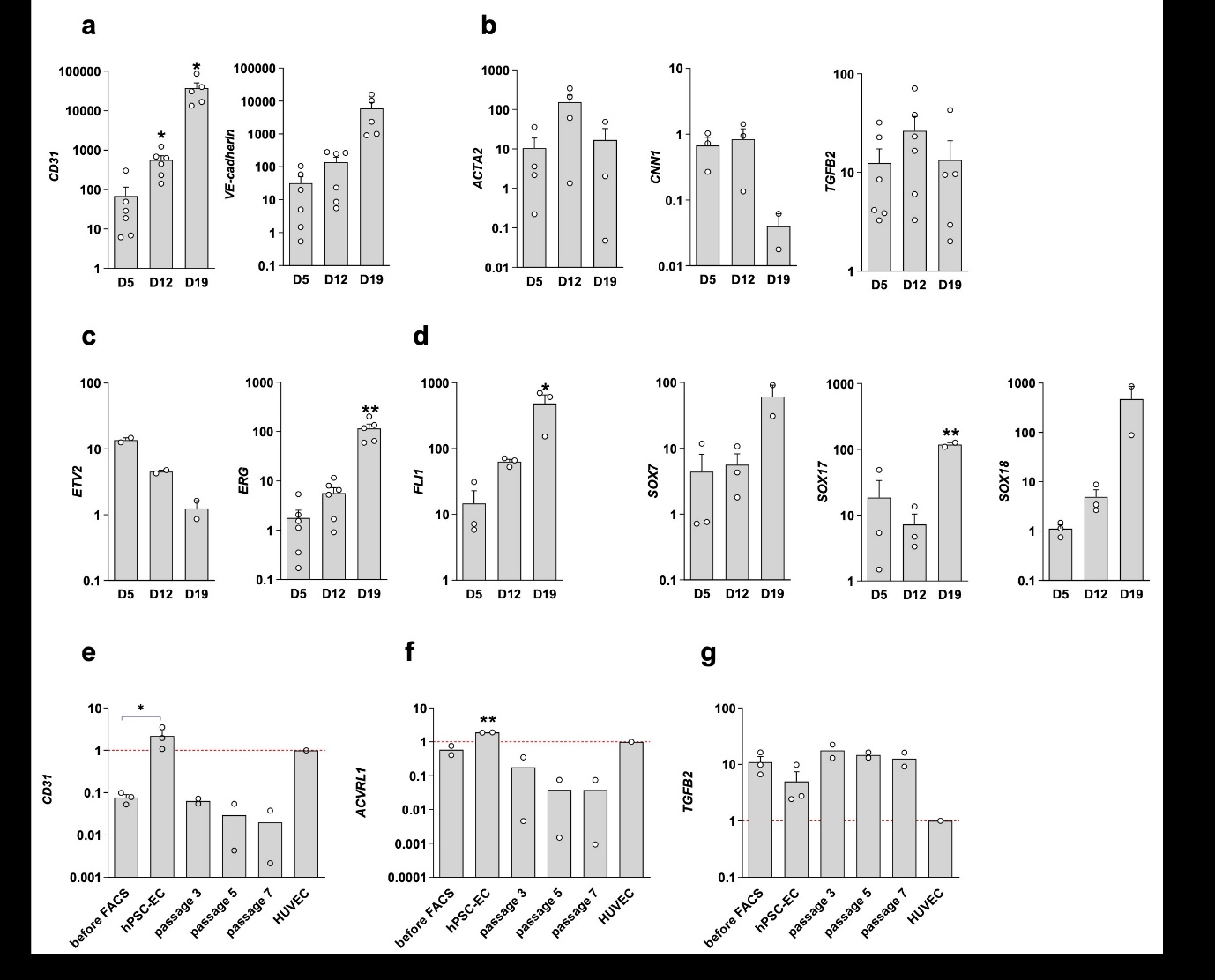


**Figure S1.** RT-qPCR analysis of **(a)** endothelial markers (*CD31* and *VE-cadherin*), **(b)** mesenchymal markers (*ACTA2, CNN1, TGFB2*), **(c)** endothelial associated transcription factors (*ETV2, ERG, FLI1*), and **(d)** SoxF transcription factors (*SOX7, SOX17, SOX18*) at day 5, day 12 (pre-FACS), and day 19 (post-FACS) of the 2D endothelial differentiation protocol. *N*=3 independent experiments, mRNA expression was determined relative to an undifferentiated hPSC control with data expressed as fold change relative to this control; note y-axis is in log scale. One-way ANOVA with Tukey’s post-hoc test: **P*<0.05; ***P*<0.01; ****P*<0.001. Expression of **(e)** *CD31,* **(f)** *ACVRL1*, and **(g)** *TGFB2* in 2D hPSC-ECs at different time points. *N*=2-3, data is represented as fold change; note y axis is in log scale. Target genes were normalised to the housekeeping gene, *GAPDH*. One-way ANOVA with Tukey’s post-hoc test: *P<0.05; **P<0.01; ***P<0.001.


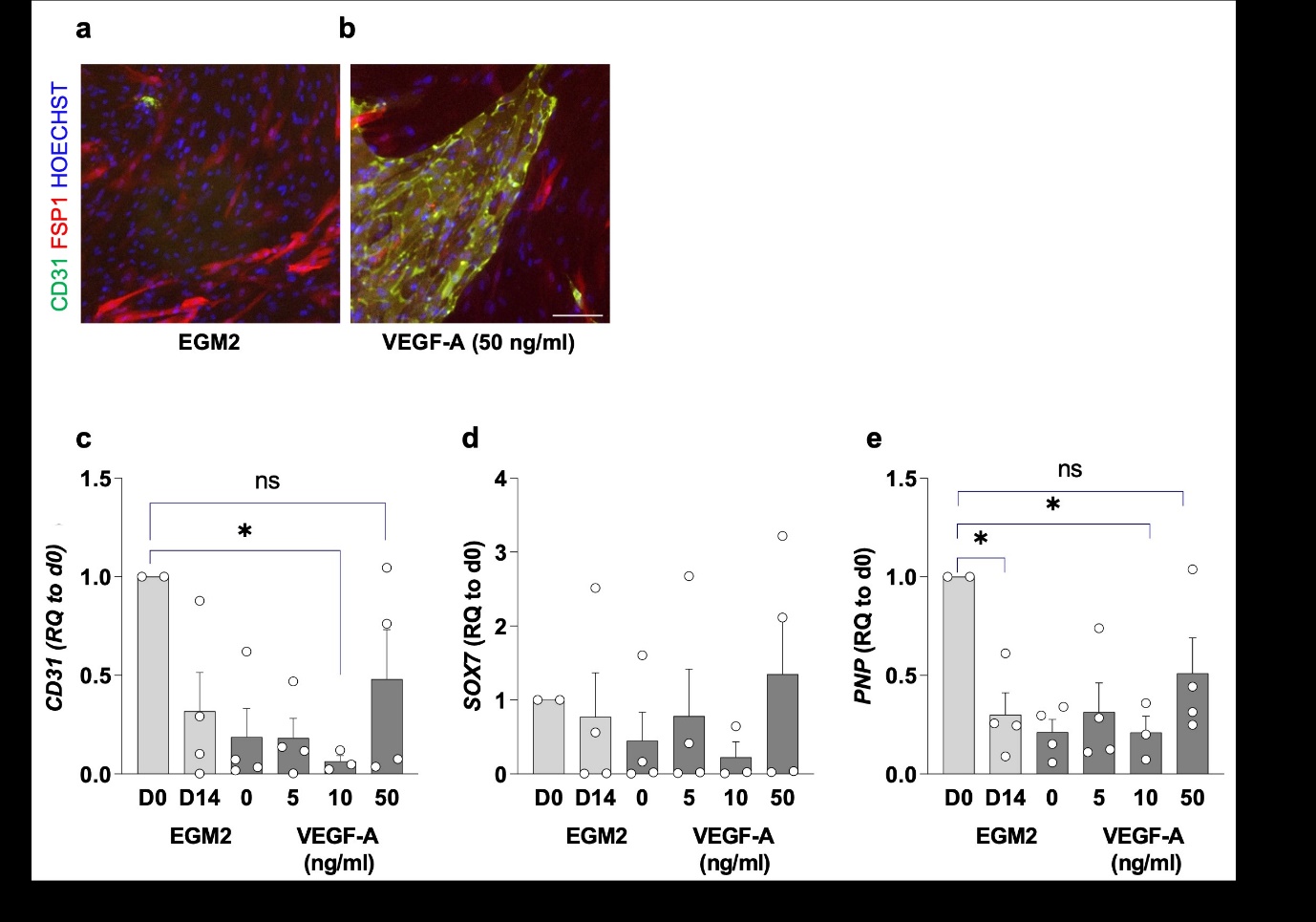


**Figure S2.** Representative immunofluorescent images of 3D hPSC-ECs that had undergone EndMT cultured in **(a)** normal EGM2 or **(b)** EGM2 supplemented with 50 ng/ml VEGF-A. CD31 (green), FSP1 (red), Hoechst (blue). Scale bars represent 50 μm. RT-qPCR analysis of **(c)** *CD31*, **(d)** *SOX7*, and **(e)** *PNP* in transdifferentiated 3D hPSC-EC cultures receiving varying concentrations of VEGF-A. *N*=3 independent experiments, data is shown as ΔΔCt, note y-axis in log scale. Target genes were normalised to the *GAPDH* housekeeping gene, one-way ANOVA with Tukey’s post-hoc test: *P<0.05.


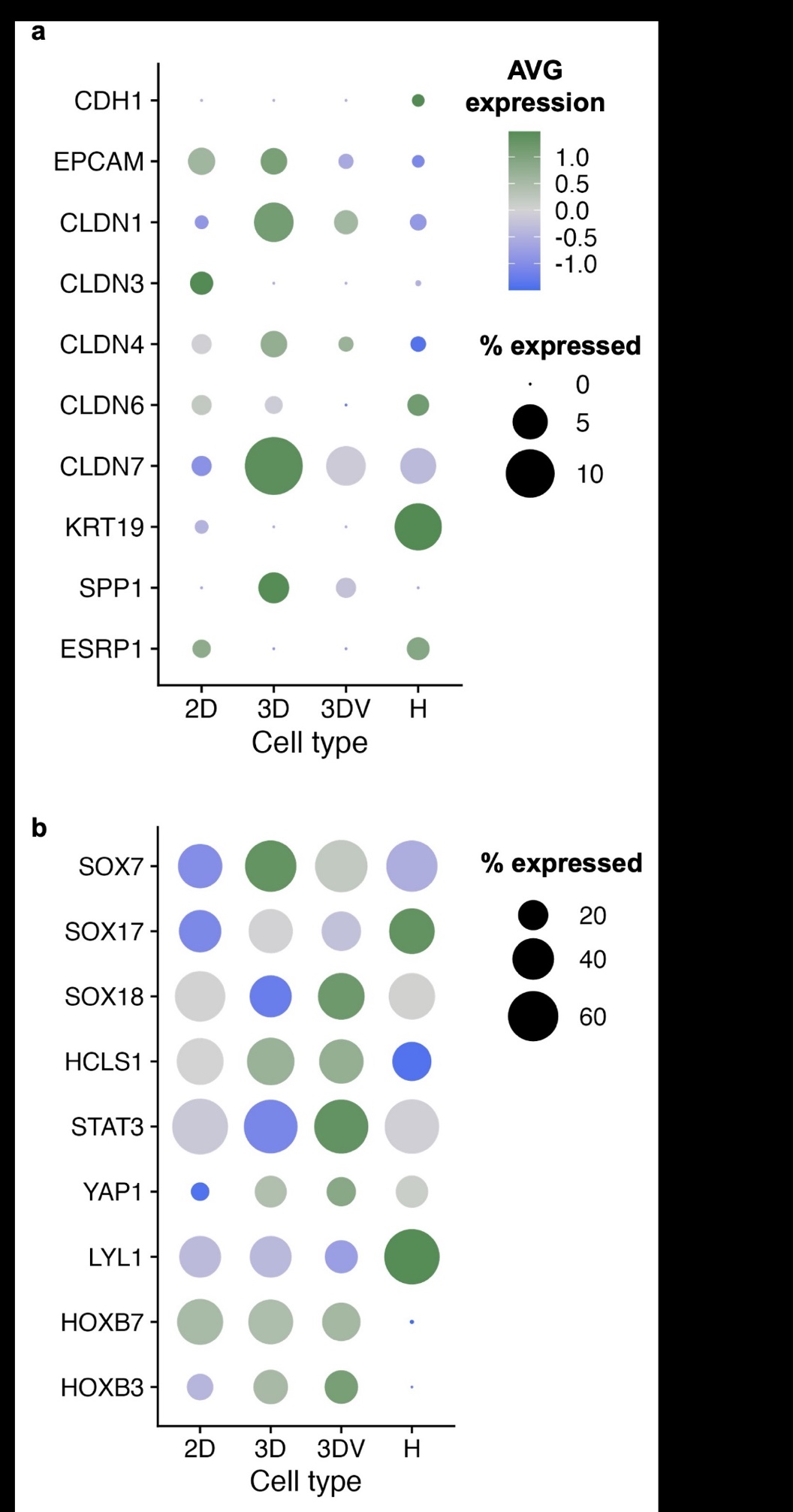


**Figure S3.** Dot plots displaying the expression levels of **(a)** epithelial-associated genes and **(b)** the CellNet-identified genes in 2D- 3D-, 3DV-hPSC-ECs, and HMVEC-Cs (referred to as ‘H’). Scale bar denoting average gene expression applies to both plots whilst the percentage of cells expressing the gene is situated next to each plot.


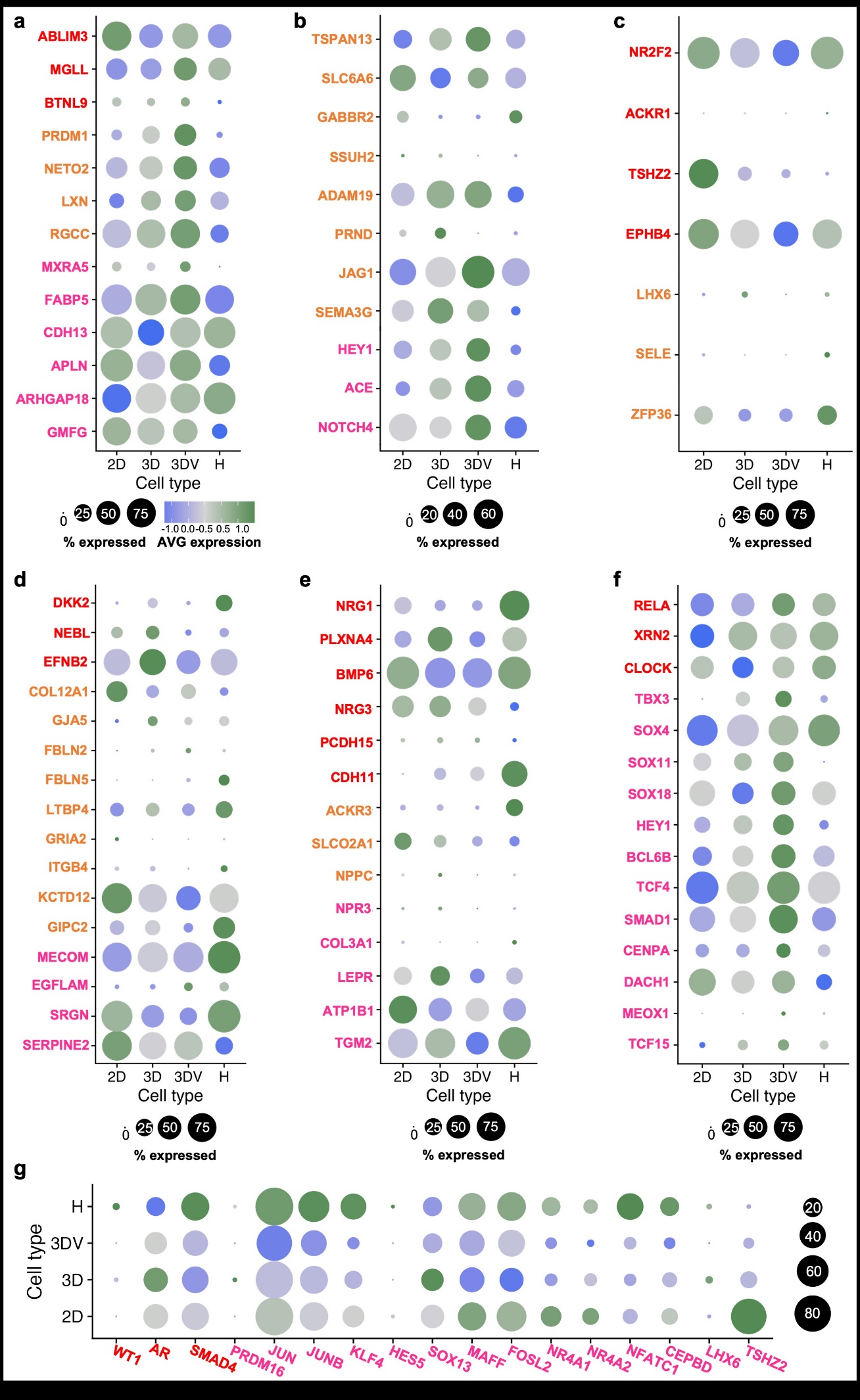


**Figure S4.** Dot plots displaying the expression levels of genes associated with cardiac- **(a)** capillaries, **(b)** arterioles, **(c)** veins, **(d)** cardiac arteries, **(e)** endocardial cells, **(f)** capillary- and microvascular-transcription factors, and **(g)** arteriovenous transcription factors in 2D- 3D-, 3DV-hPSC-ECs, and HMVEC-Cs (referred to as ‘H’). Scale bar denoting average gene expression is situated under ‘a’ and applies to ‘a-g’ whilst the percentage of cells expressing the gene is placed under each plot. The colour of the gene name refers to the publication in which it was reported to be expressed in human cardiac endothelial cells: Koenig AL *et al.* (red), Phansalkar R et al. (orange), and McCracken IR et al. (pink).


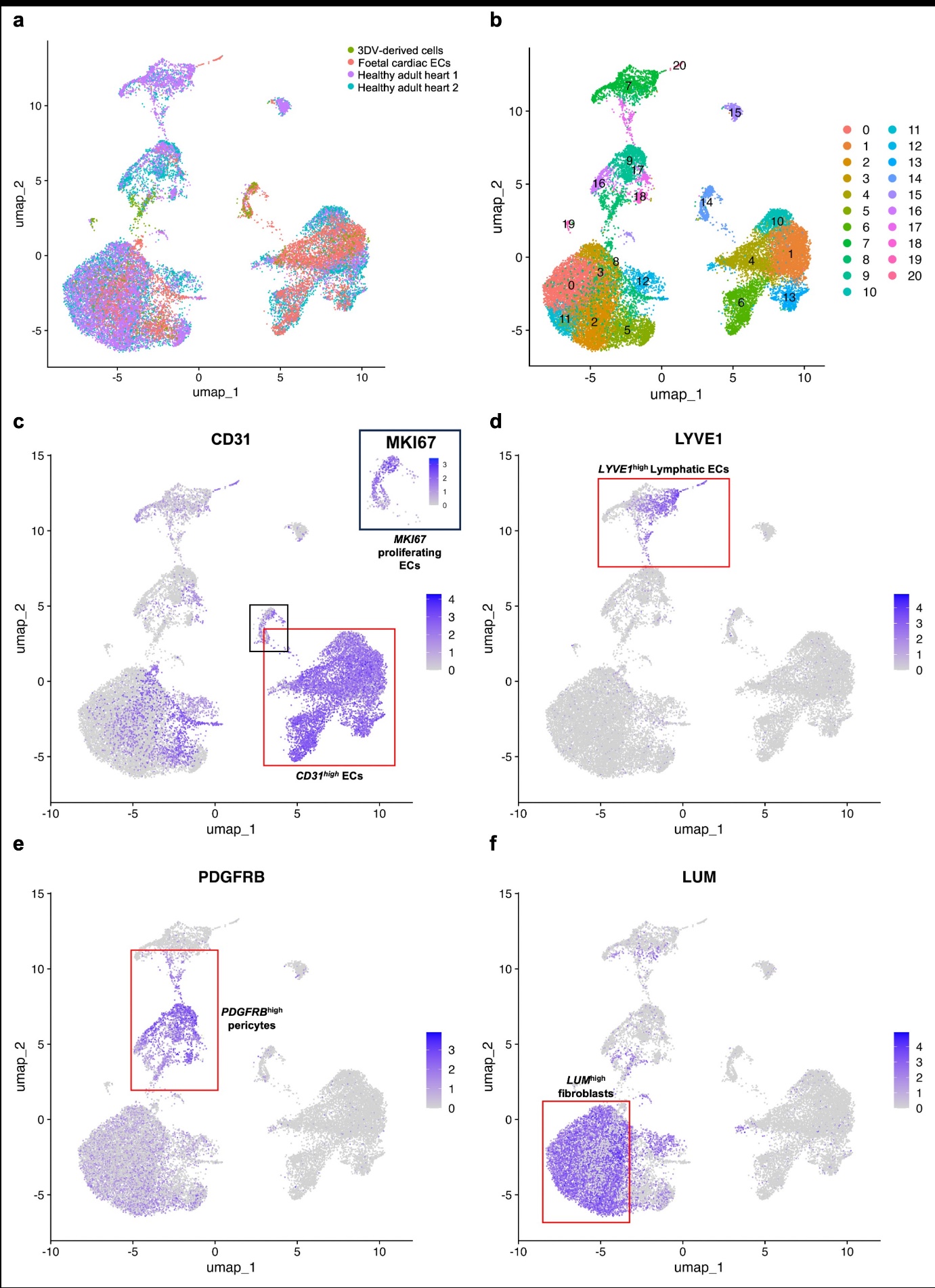


**Figure S5.** UMAP plots emerging from **(a)** the integration of 3DV-derived cells (green), with published foetal cardiac ECs (red), and two published healthy adult heart samples (cyan and purple) and **(b)** the resulting clusters within this complete integrated data set. Feature plots for **(c)** *CD31 (*black inset: proliferation marker *MKI67*), **(d)** *LYVE1,* **(e)** *PDGFRB*, and **(f)** *LUM* expression within this integrated data set. Cells demonstrating high expression of these marker genes are highlighted within the red box. Scale bars denoting the average gene expression are situated to the right of the respective feature plot.


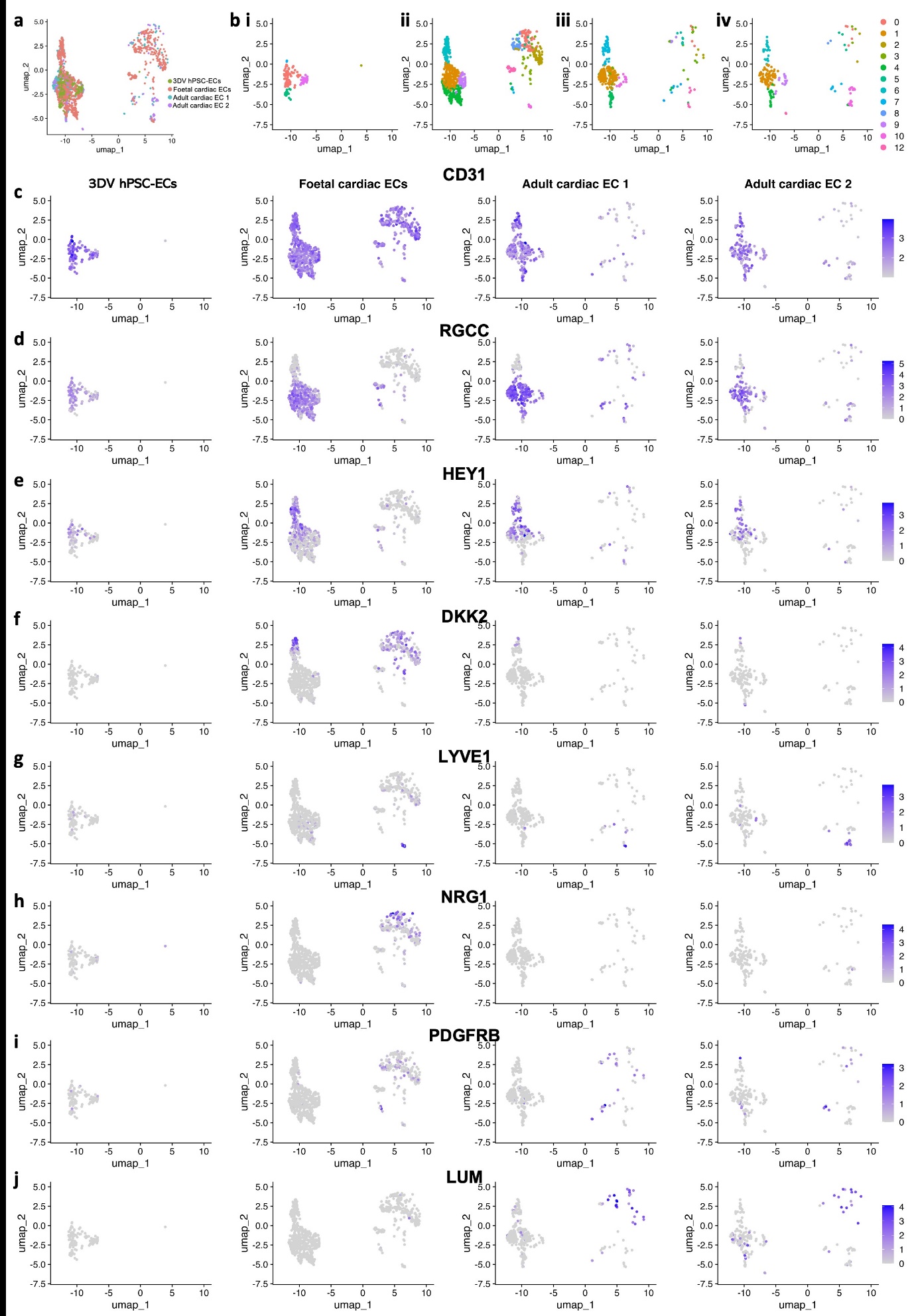


**Figure S6.** UMAP plots illustrating **(a)** the integrated data set following downsampling of published samples and selection of *CD31*^high^ cells from all samples, and **(b)** the resulting clusters displayed by the (i) 3DV hPSC-ECs, (ii) foetal cardiac ECs, (iii) adult cardiac EC 1, and (iv) adult cardiac EC 2. Note, upon selection of the *CD31*^high^ cells from the healthy adult heart samples, these are now designated as adult cardiac EC 1 and 2, respectively. Feature plots for **(c)** *CD31,* **(d)** *RGCC*, **(e)** *HEY1*, **(f)** *DKK2*, **(g)** *LYYVE1*, **(h)** *NRG1*, **(i)** *PDGFRB*, and **(j)** *LUM* expression across the four samples. Scale bars denoting the average gene expression are situated to the right of the respective feature plot.


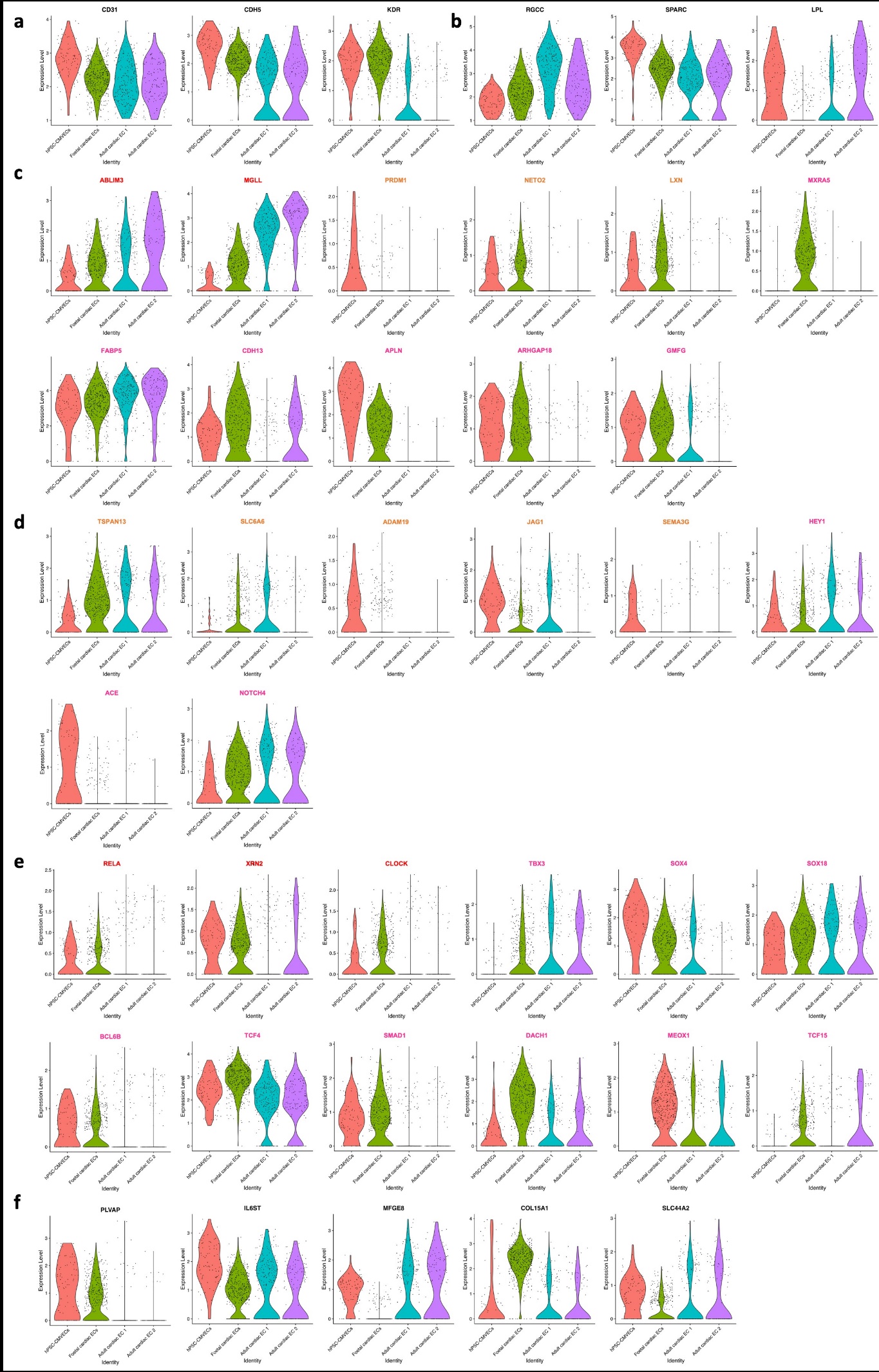


**Figure S7.** Violin plots displaying the expression levels of genes associated with **(a)** endothelial cells, **(b)** capillary ECs, **(c)** cardiac capillaries, **(d)** cardiac arterioles, **(e)** cardiac capillary and microvascular-transcription factors, and **(f)** cardiac endothelial-associated genes within the *CD31*^high^/*RGCC*^high^ cells across the four samples: hPSC-CMVECs (red), foetal cardiac ECs (green), and adult cardiac EC 1 (cyan) and 2 (purple). The colour of the gene name in ‘c-e’ refers to the publication in which it was reported to be expressed in human cardiac endothelial cells: Koenig AL *et al.* (red), Phansalkar R et al. (orange), and McCracken IR et al. (pink).


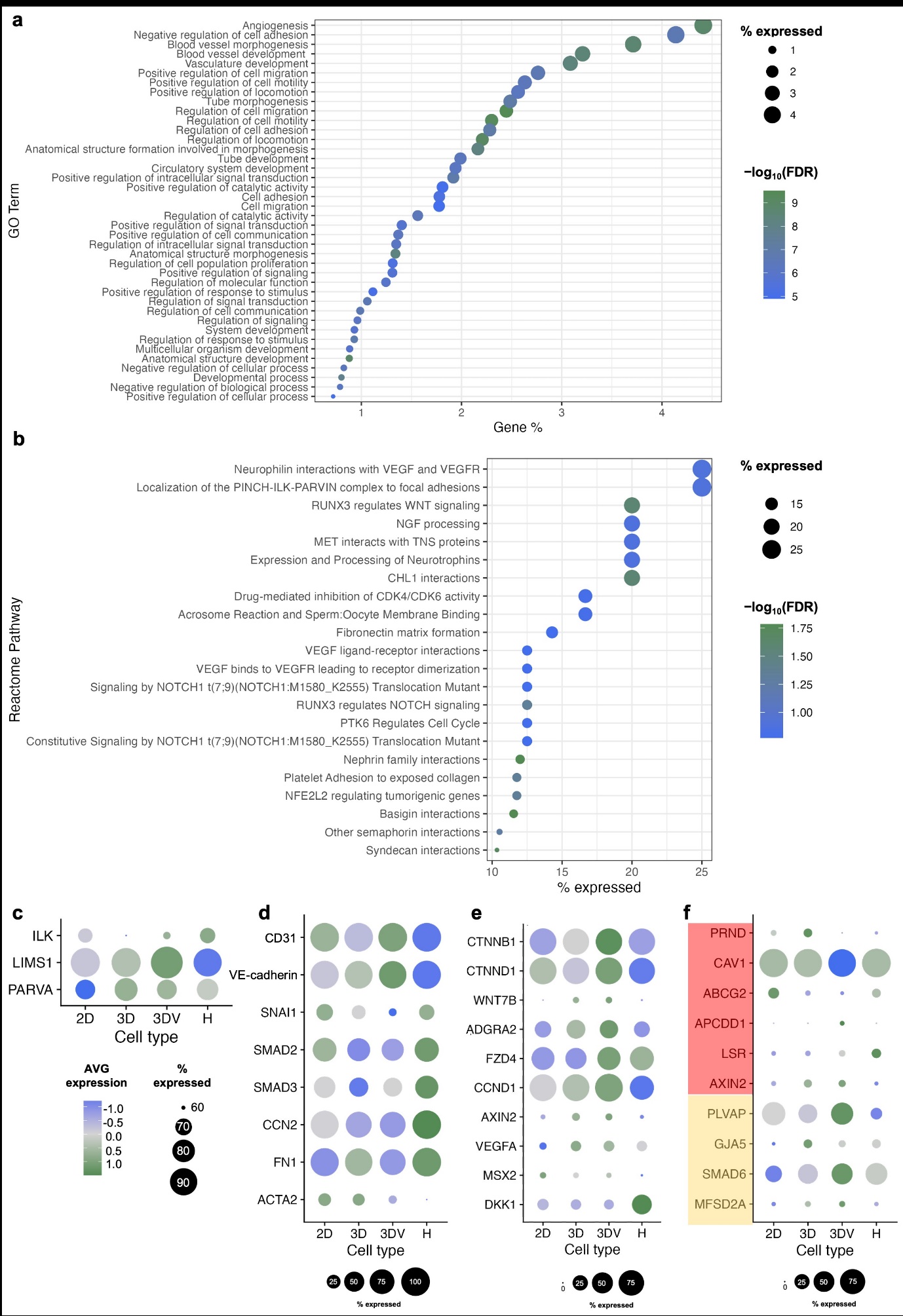


**Figure S8. (a)** Gene Ontology (GO) enrichment analysis and **(b)** Reactome Pathway analysis emerging from total DEGs identified in 3DV hPSC-ECs relative to 3D hPSC-ECs. The top 40 GO terms, as determined by their false discovery rate (FDR), are displayed. The scales denote the percentage of genes expressed per GO term or Reactome pathway relative to the reference list for the particular term or pathway and the -log_10_FDR. Dot plots displaying the expression levels of genes associated with the: **(c)** PINCH-ILK-PARVIN complex, *LIMS1* and *PARVA* encode for PINCH and PARVIN, respectively, **(d)** anti-EndMT effects of ILK, **(e)** WNT7 signalling, and **(f)** blood-brain barrier (BBB) properties in 2D- 3D-, 3DV-hPSC-ECs, and HMVEC-Cs (referred to as ‘H’). Note the red and yellow boxes in ‘f’ that denote genes most upregulated or downregulated in the BBB model, respectively. Scale bar denoting average gene expression is situated under ‘c’ and applies to ‘c-f’ whilst the percentage of cells expressing the gene is listed under each plot.


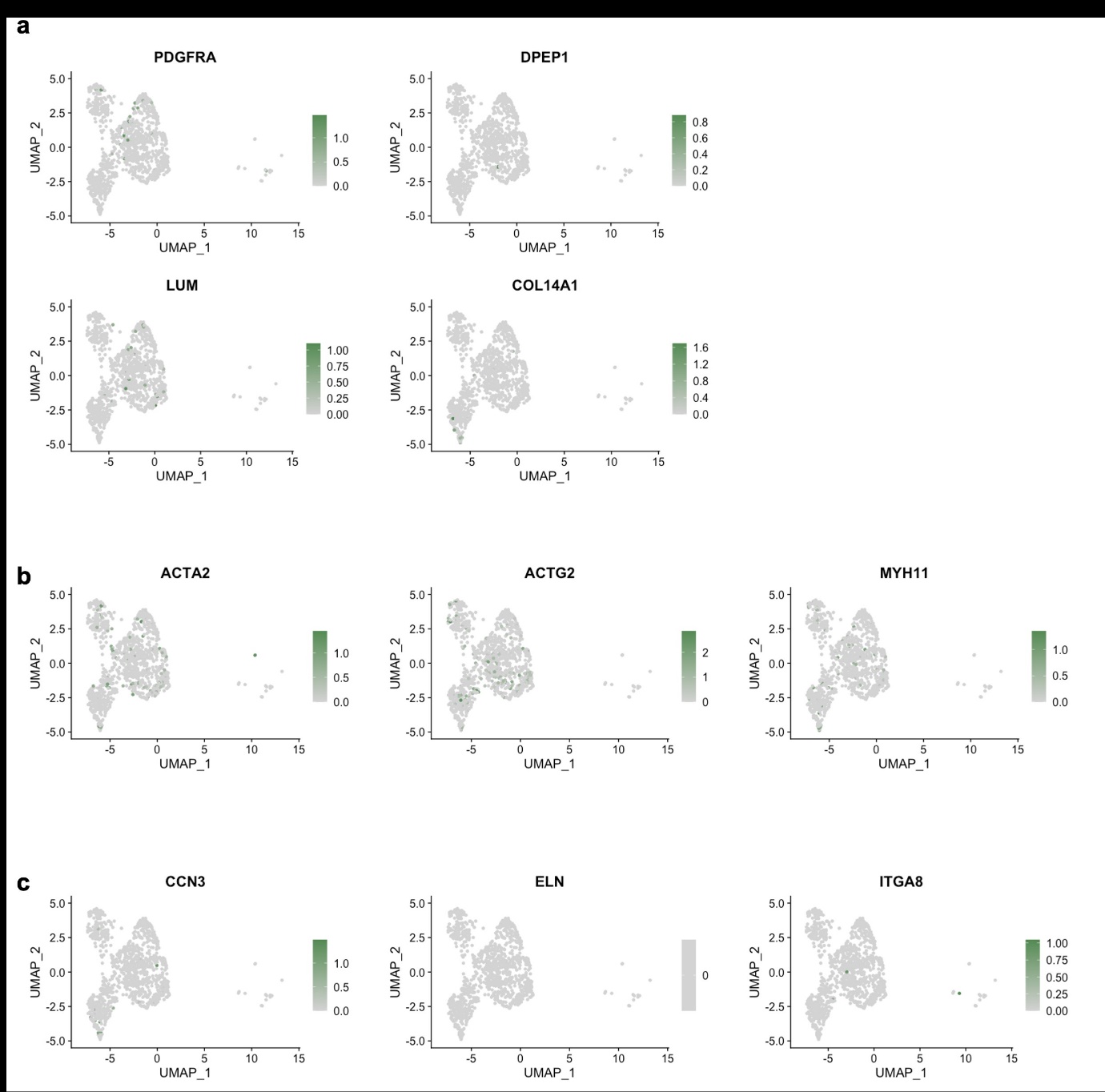


**Figure S9.** UMAP plots of the *CD31*^neg^ population of cells arising from the vascular organoids showcasing expression of **(a)** fibroblast-specific genes, and vascular smooth muscle cell (vSMC) genes identified in vSMCs either **(b)** across organs or **(c)** within large coronary vessels. Scale bars illustrating average expression are placed next to each plot.


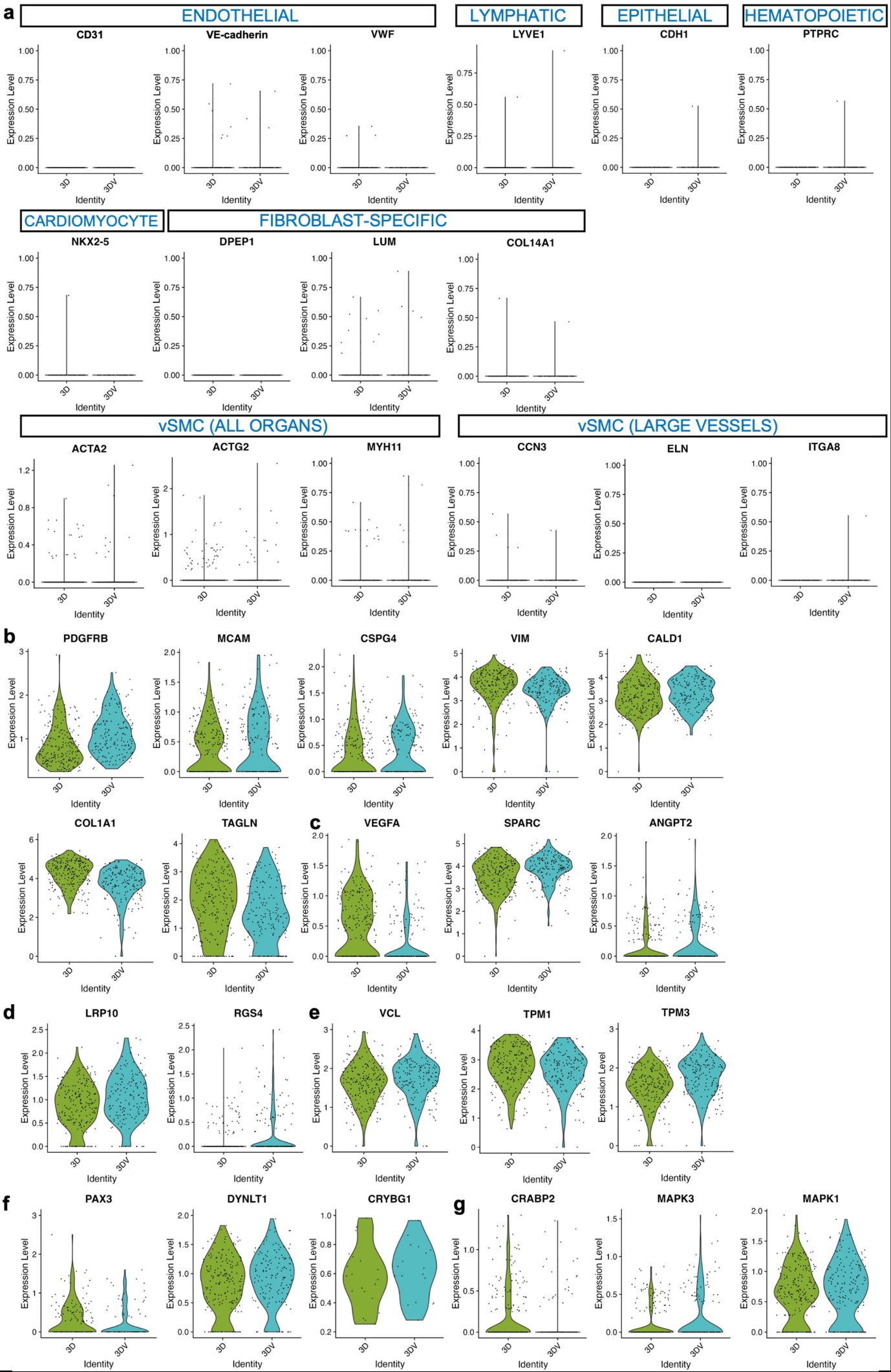


**Figure S10.** Violin plots of the *CD31*^neg^/*PDGFRB*^pos^ population of cells derived from 3D (green) and 3DV (cyan) cultures displaying expression of genes associated with: **(a)** non-pericytes, **(b)** pericytes, **(c)** factors released by pericytes, **(d)** cardiac-pericyte specificity, either **(e)** upregulation or **(f)** preferential expression relative to vSMCs, and **(g)** differentiation of cardiac pericytes into vSMCs.
